# A comparison of conventional and resampled personal reliability in detecting careless responding

**DOI:** 10.3758/s13428-024-02506-0

**Published:** 2024-09-16

**Authors:** Philippe Goldammer, Peter Lucas Stöckli, Hubert Annen, Annika Schmitz-Wilhelmy

**Affiliations:** 1 Military Academy at ETH Zurich, Birmensdorf, Switzerland; 2 Department of Psychology, University of Zurich, Zurich, Switzerland

**Keywords:** Careless responding detection, Even–odd consistency, Personal reliability, Resampled personal reliability

## Abstract

Detecting careless responding in survey data is important to ensure the credibility of study findings. Of several available detection methods, personal reliability (PR) is one of the best-performing indices. Curran, *Journal of Experimental Social Psychology, 66*, 4-19, (2016) proposed a resampled version of personal reliability (RPR). Compared to the conventional PR or even–odd consistency, in which just one set of scale halves is used, RPR is based on repeated calculation of PR across several randomly rearranged sets of scale halves. RPR should therefore be less affected than PR by random errors that may occur when a specific set of scale half pairings is used for the PR calculation. In theory, RPR should outperform PR, but it remains unclear whether it in fact does, and under what conditions the potential gain in detection accuracy is the most pronounced. We conducted two studies: a simulation study examined the performance of the conventional PR and RPR in detecting simulated careless responding, and a real data example study analyzed their performance when detecting human-generated careless responding. In both studies, RPR turned out to be a significantly better careless response indicator than PR. The results also revealed that using 25 resamples for the RPR computation is sufficient to obtain the expected gain in detection accuracy over the conventional PR. We therefore recommend using RPR instead of the conventional PR when screening questionnaire data for careless responding.

**Supplementary Information:**

The online version contains supplementary material available at 10.3758/s13428-024-02506-0.

Likert scale questionnaires are a convenient and routinely used method to assess different psychological phenomena. However, credible results can be obtained by this method only if respondents have answered the questions attentively. Unfortunately, it is rather common that some respondents in the sample rush through the questionnaire without paying attention to the item content and instructions (Goldammer et al., 2020; Meade & Craig, 2012)―a response behavior that has been commonly labeled insufficient effort responding (Huang et al., 2012) or careless responding (Meade & Craig, 2012).

Undetected careless responding can have severe consequences. Simulation studies suggest that even small proportions of careless respondents in the data, 10% (Hong et al., 2020; Woods, 2006) or even only 5% (Credé, 2010), can bias results. Moreover, the bias increases with the rate of careless respondents (Hong et al., 2020). In addition, careless responding has a biasing effect on a variety of estimates, such as item covariances (Credé, 2010; Goldammer et al., 2020), item means (Goldammer et al., 2020), reliability estimates (Hong et al., 2020; Huang et al., 2012), factor loadings (Kam & Meyer, 2015; Meade & Craig, 2012), and the testing of construct dimensionality (Arias et al., 2020; Goldammer et al., 2020; Woods, 2006). It is not surprising that research on detecting careless responding has gained considerable attention and that several detection methods have been proposed (for an overview, see Curran, 2016).

## Personal reliability

Among the proposed methods, personal reliability (PR; Jackson, 1976), which has also been called individual reliability or even–odd consistency (Curran, 2016; DeSimone et al., 2015), has turned out to be one of the best-performing indices in detecting careless responding (Goldammer et al., 2020, 2024; Huang et al., 2012; Meade & Craig, 2012; Niessen et al., 2016). PR makes use of a very simple logic: Careful respondents should not contradict themselves over the course of a questionnaire and are expected to choose similar response options when they rate items from the same unidimensional scale. Accordingly, a score that is based on half the scale items (e.g., even-numbered items) should correlate positively with a score based on the rest of the scale items (e.g., odd-numbered items). If a questionnaire includes at least three such pairs of scale halves, PR may then be computed as within-person correlation across the two vectors of scale half pairings (Curran, 2016; DeSimone et al., 2015).

The computational principle of PR may be further illustrated with a simplified example. Let us assume that researchers have gathered data for two persons who completed a personality questionnaire measuring the five broad personality dimensions openness, conscientiousness, extraversion, agreeableness, and emotional stability, and that each of the five broad dimensions was measured with four items on a unidirectionally keyed Likert scale with six response options. Table 1 shows the simulated response protocols for these two persons. The protocol of person 1 was simulated such that it mimics a careful response pattern,^1^ and the protocol of person 2 was simulated such that it mimics a careless (i.e., random uniform) response pattern.

**Table 1:** Simulated response protocols of a careful and a careless responder to a 20-item questionnaire

Item	Person 1 (careful responder)	Person 2 (careless responder)
OP1	4	6
OP2	3	5
OP3	4	1
OP4	4	5
CO1	4	3
CO2	3	2
CO3	4	5
CO4	4	1
EX1	3	4
EX2	3	1
EX3	3	2
EX4	3	5
AG1	2	6
AG2	3	5
AG3	3	1
AG4	3	6
EM1	3	1
EM2	4	1
EM3	3	1
EM4	4	5

*Note*. OP = openness; CO = conscientiousness; EX = extraversion; AG = agreeableness; EM = emotional stability

To obtain PR, the researchers proceed as follows. First, they build halves for each of the five dimensions and calculate an average score for these halves. In our example, the researchers calculated scores for the even-numbered and the odd-numbered items and rearranged the data from wide to long format, such that each person has five row entries and the two scale halves are represented as two separate columns or vectors (see Table 2). Based on this data format, PR can be calculated for each person by correlating the two vectors of scale halves. If desired, these PR values can also be corrected with the Spearman–Brown prophesy formula (${\text{PR}}_{\text{SB}}= \frac{(\text{k}*\text{PR})}{(1+\left(\text{k}-1\right)*\text{PR})}$; Brown, 1910; Spearman, 1910), in which *k* is the factor of scale reduction (in our case, *k* equals 2). In our example, the researchers would have obtained a PR_SB_ of .525 for person 1 and a PR_SB_ of .178 for person 2. Based on these PR_SB_ values, the researchers then conclude (unaware of our data-generating process) that person 2 has responded on the personality questionnaire and its scales in a far more inconsistent manner than person 1. After consulting the literature for commonly applied PR cut scores (e.g., below .3; DeSimone et al., 2015; Zickar & Keith, 2023), the researchers would have even begun to doubt the responding effort of person 2.

**Table 2:** Scale scores of even- and odd-numbered items and resulting personal reliability values for person 1 (careful responder) and person 2 (careless responder)

Participant	Scores of even-numbered items	Scores of odd-numbered items
Person 1	OP (3_Item 2_ + 4_Item 4_) / 2 = 3.5	OP (4_Item 1_ + 4_Item 3_) / 2 = 4.0
Person 1	CO (3_Item 2_ + 4_Item 4_) / 2 = 3.5	CO (4_Item 1_ + 4_Item 3_) / 2 = 4.0
Person 1	EX (3_Item 2_ + 3_Item 4_) / 2 = 3.0	EX (3_Item 1_ + 3_Item 3_) / 2 = 3.0
Person 1	AG (3_Item 2_ + 3_Item 4_) / 2 = 3.0	AG (2_Item 1_ + 3_Item 3_) / 2 = 2.5
Person 1	EM (4_Item 2_ + 4_Item 4_) / 2 = 4.0	EM (3_Item 1_ + 3_Item 3_) / 2 = 3.0
PR_SB_ Person 1	.525	
Person 2	OP (5_Item 2_ + 5_Item 4_) / 2 = 5.0	OP (6_Item 1_ + 1_Item 3_) / 2 = 3.5
Person 2	CO (2_Item 2_ + 1_Item 4_) / 2 = 1.5	CO (3_Item 1_ + 5_Item 3_) / 2 = 4.0
Person 2	EX (1_Item 2_ + 5_Item 4_) / 2 = 3.0	EX (4_Item 1_ + 2_Item 3_) / 2 = 3.0
Person 2	AG (5_Item 2_ + 6_Item 4_) / 2 = 5.5	AG (6_Item 1_ + 1_Item 3_) / 2 = 3.5
Person 2	EM (1_Item 2_ + 5_Item 4_) / 2 = 3.0	EM (1_Item 1_ + 1_Item 3_) / 2 = 1.0
PR_SB_ Person 2	.178	

*Note*. OP = openness; CO = conscientiousness; EX = extraversion; AG = agreeableness; EM = emotional stability; PR_SB_ = personal reliability (i.e., even–odd consistency) corrected with Spearman–Brown formula

### Resampled personal reliability

Calculating the PR by using even–odd scale half pairs has been the standard for many years (Curran, 2016, pp. 9–10). Nevertheless, it is an arbitrary choice for building the scale halves that may be as good or bad as any other set of scale halves. To overcome this arbitrariness, Curran (2016, pp. 9–10) therefore proposed using a resampled version of personal reliability, which he called resampled individual reliability (RIR) (here, resampled personal reliability, RPR). This entails calculating PR with different sets of scale halves and summarizing the results to an overall PR measure, for instance by building the arithmetic mean of the PR values (i.e., computing a mean-based RPR).^2^

The principle of the RPR may be again illustrated using our simulated response protocols. In Table 3 we show the scores of three different random sets of scale half pairings (out of 3^5^ potential pairs) and the resulting PR and RPR values for carefully responding person 1 and carelessly responding person 2. Based on the PR and RPR values in Table 3, the following observations and conclusions can be made. First, the PRs vary depending on the sets of scale half pairings that were used for computation, from .811 to .866 for person 1 and from –.472 to .263 for person 2 (Table 3).^3^ Second, if researchers use only a single set of scale half pairings, they may be unlucky with their choice and end up with a PR measure that only poorly reflects the true state of the respondent’s responding effort. For instance, if PR had been calculated based only on the second random split of items, the researchers would have obtained a PR_SB_ of .263 for person 2 and in turn may have not doubted the person’s responding effort, as they should have or would have when using another set of scale half pairings. Third, by taking the average of PR values that were calculated across different sets of scale half pairings (and thus calculating RPR), the researchers may obtain not only a measure that is less arbitrary in its computation but also one that is less affected by “sampling error” (i.e., fluctuation in PR values that occurs when using different sets of scale half pairings). Eventually, this reduced amount of sampling error in RPR is also why RPR has been suspected to be a more precise careless response indicator than the conventional PR measure (Curran, 2016, pp. 9–10; Ward & Meade, 2023, p. 587), which is based on only a single set of scale half pairings—typically a set of even–odd scale half pairs.

**Table 3:** Scale scores of different sets of scale half pairings (i.e., splits) and resulting personal reliability values for person 1 (careful responder) and person 2 (careless responder)

	First random split of items		Second random split of items		Third random split of items		
Participant	Scores first half	Scores second half	Scores first half	Scores second half	Scores first half	Scores second half	RPR3
Person 1	OP (3_Item 1_ + 4_Item 2_) / 2 = 3.5	OP (4_Item 3_ + 4_Item 4_) / 2 = 4.0	OP (4_Item 3_ + 4_Item 4_) / 2 = 4.0	OP (4_Item 1_ + 3_Item 2_) / 2 = 3.5	OP (4_Item 1_ + 4_Item 3_) / 2 = 4.0	OP (3_Item 2_ + 4_Item 4_) / 2 = 3.5	
Person 1	CO (3_Item 2_ + 4_Item 4_) / 2 = 3.5	CO (4_Item 1_ + 4_Item 3_) / 2 = 4.0	CO (3_Item 2_ + 4_Item 4_) / 2 = 3.5	CO (4_Item 1_ + 4_Item 3_) / 2 = 4.0	CO (3_Item 2_ + 4_Item 4_) / 2 = 3.5	CO (4_Item 1_ + 4_Item 3_) / 2 = 4.0	
Person 1	EX (3_Item 2_ + 3_Item 4_) / 2 = 3.0	EX (3_Item 1_ + 3_Item 3_) / 2 = 3.0	EX (3_Item 1_ + 3_Item 2_) / 2 = 3.0	EX (3_Item 3_ + 3_Item 4_) / 2 = 3.0	EX (3_Item 1_ + 3_Item 4_) / 2 = 3.0	EX (3_Item 2_ + 3_Item 3_) / 2 = 3.0	
Person 1	AG (2_Item 1_ + 3_Item 3_) / 2 = 2.5	AG (3_Item 2_ + 3_Item 4_) / 2 = 3.0	AG (3_Item 3_ + 3_Item 4_) / 2 = 3.0	AG (2_Item 1_ + 3_Item 2_) / 2 = 2.5	AG (2_Item 1_ + 3_Item 2_) / 2 = 2.5	AG (3_Item 3_ + 3_Item 4_) / 2 = 3.0	
Person 1	EM (3_Item 1_ + 3_Item 3_) / 2 = 3.0	EM (4_Item 2_ + 4_Item 4_) / 2 = 4.0	EM (3_Item 1_ + 4_Item 4_) / 2 = 3.5	EM (4_Item 2_ + 3_Item 3_) / 2 = 3.5	EM (3_Item 1_ + 4_Item 2_) / 2 = 3.5	EM (3_Item 3_ + 4_Item 4_) / 2 = 3.5	
PR_SB_ Person 1	.866		.811		.811		
RPR3 Person 1							.83
Person 2	OP (6_Item 1_ + 5_Item 2_) / 2 = 5.5	OP (1_Item 3_ + 5_Item 4_) / 2 = 3.0	OP (1_Item 3_ + 5_Item 4_) / 2 = 3.0	OP (6_Item 1_ + 5_Item 2_) / 2 = 5.5	OP (6_Item 1_ + 1_Item 3_) / 2 = 3.5	OP (5_Item 2_ + 5_Item 4_) / 2 = 5.0	
Person 2	CO (2_Item 2_ + 1_Item 4_) / 2 = 1.5	CO (3_Item 1_ + 5_Item 3_) / 2 = 4.0	CO (2_Item 2_ + 1_Item 4_) / 2 = 1.5	CO (3_Item 1_ + 5_Item 3_) / 2 = 4.0	CO (1_Item 2_ + 2_Item 4_) / 2 = 1.5	CO (3_Item 1_ + 5_Item 3_) / 2 = 4.0	
Person 2	EX (1_Item 2_ + 5_Item 4_) / 2 = 3.0	EX (4_Item 1_ + 2_Item 3_) / 2 = 3.0	EX (4_Item 1_ + 1_Item 2_) / 2 = 2.5	EX (2_Item 3_ + 5_Item 4_) / 2 = 3.5	EX (4_Item 1_ + 5_Item 4_) / 2 = 4.5	EX (1_Item 2_ + 2_Item 3_) / 2 = 1.5	
Person 2	AG (6_Item 1_ + 1_Item 3_) / 2 = 3.5	AG (5_Item 2_ + 6_Item 4_) / 2 = 5.5	AG (1_Item 3_ + 6_Item 4_) / 2 = 3.5	AG (6_Item 1_ + 5_Item 2_) / 2 = 5.5	AG (6_Item 1_ + 5_Item 2_) / 2 = 5.5	AG (1_Item 3_ + 6_Item 4_) / 2 = 3.5	
Person 2	EM (1_Item 1_ + 1_Item 3_) / 2 = 1.0	EM (1_Item 2_ + 5_Item 4_) / 2 = 3.0	EM (1_Item 1_ + 5_Item 4_) / 2 = 3.0	EM (1_Item 2_ + 1_Item 3_) / 2 = 1.0	EM (1_Item 1_ + 1_Item 2_) / 2 = 1	EM (1_Item 3_ + 5_Item 4_) / 2 = 3.0	
PR_SB_ Person 2	.025		.263		–.472		
RPR_3_ Person 2							–.06

*Note*. OP = openness; CO = conscientiousness; EX = extraversion; AG = agreeableness; EM = emotional stability; PR_SB_ = personal reliability corrected with Spearman–Brown formula; RPR3 = resampled personal reliability based on three random scale half splits (i.e., average of the three PR_SB_)

### Purpose of current studies and research questions

Unfortunately, up to now, no study that we know of has systematically examined whether RPR really outperforms PR and under what conditions the potential gain in detection accuracy is the most pronounced (e.g., Zickar & Keith, 2023). For this reason, we conducted a simulation study, and we reanalyzed the data of Goldammer et al.’s (2020) experimental study on careless responding. The simulation study allowed us to examine the performance of PR and RPR across a multitude of conditions that could not be easily set up in a real experiment. However, a simulation study also entails using simulated careless responses protocols, which may only partially reflect human-generated careless response protocols. Niessen et al. (2016), for instance, found the detection rates of several careless response indices to be remarkably lower when human-generated careless response data were used than when computer-generated random data were used. Reanalyzing Goldammer et al.’s (2020) experimental data therefore allowed us to examine the utility of PR and RPR under conditions that are closer to real human careless responding in surveys and thus under conditions in which careless responding may be harder to detect. In sum, our two studies therefore address the following three research questions:

- ***Research question 1:*** Does RPR outperform the conventional PR (i.e., even–odd consistency)?
- ***Research question 2:*** Under what conditions is the gain in detection accuracy that is expected when using RPR instead of PR the most pronounced?
- ***Research question 3:*** Does the performance of RPR and PR differ when human instead of computer-generated careless response patterns need to be detected?

## The simulation study

### General design

To generate the data in our simulation study, we used latent factor models (Jöreskog, 1969), in which an observed item response can be described as a function of the underlying latent factor, the latent factor’s loading on the observed item, and an item-specific error variance. We selected this model type because it allowed us to simulate response protocols of multi-facet surveys and to manipulate parameters that we thought were most likely to have an effect on the detection accuracy of PR and/or RPR. In sum, we examined the performance of PR and RPR across (3*3*2*2) 36 conditions with 100 replications for each condition. For all these data-generating processes, we used Stata 18 (StataCorp, 2023). Table 4 provides an overview of the manipulated and fixed parameters in our simulation study.

**Table 4:** Design of the simulation study

Parameters	Levels
Manipulated	
Number of facets (latent factors)	5; 15; 30
Extent of item-specific error per facet (latent factor)	Low item-specific error per facet: Error variances were low in all items measuring each facet (i.e., normally distributed errors with *N*[0, 0.5] for all items of each factor) Mediocre item-specific error per facet: Error variances were low for one half of the items of each facet (i.e., normally distributed errors with *N*[0, 0.5]) and high for the other half of the items of each facet (i.e., normally distributed errors with *N*[0, 1.5]) High item-specific error per facet: Error variances were high in all items measuring each facet (i.e., normally distributed errors with *N*[0, 1.5] for all items of each factor)
Careless responding type	Uniform random; invariant
Careless responding severity	Full (all item responses of the response protocol are simulated to be given carelessly); partial (50% of the item responses of the response protocol are simulated to be given carelessly)
Fixed	
Number of observations	300
Percentage of careless responders in the sample	30%
Facet (latent factor) correlation	.3
Facet (latent factor) distribution	Normally distributed latent factors with *N*(3.5, 0.7)
Items per facet (latent factor)	4
Item keying	Unidirectional positive keying for all items
Facet (latent factor) loading for each item	1
Item response format	6-point scale

### Manipulated parameters

We manipulated four parameters in our simulation study. First, we examined the performance of PR and RPR across three facet (latent factor) sample sizes: 5, 15, and 30. Because the number of items was fixed at 4 per facet, the item sample sizes for the three facet conditions were as follows: 20, 60, 120. These facet (and item) sample sizes were chosen because they reflected the facet (and item) sample sizes of typical short and comprehensive personality questionnaires (Donnellan et al., 2006; Johnson, 2014; Soto & John, 2017). The condition with five facets may be taken as an example of a typical short personality questionnaire in which only five facets are assessed, such as the Mini-IPIP (Donnellan et al., 2006), which has 20 items measuring five broad personality traits. Furthermore, the condition with five facets represents a situation in which the number of available facets was only barely above the required minimum (i.e., 3) that is necessary to calculate the PR measures. In contrast, the conditions with 15 and 30 facets were clearly above the required minimum of facets. These conditions may be taken as examples of comprehensive personality questionnaires in which several facets are assessed, such as the Big Five Inventory-2 (Soto & John, 2017), which has 60 items measuring 15 facets, or the IPIP-NEO-120 (Johnson, 2014), with 120 items measuring 30 facets. Because the number of facets (or scale half pairings) acts as sample size when computing personal reliability measures, more precise careless responding estimates can be expected when more facets and thus larger sample sizes are used (see DeSimone et al., 2015, p. 175). We expected PR and the RPR to be more accurate indicators of careless responding when 15 or even 30 facets were used for computation than when only five facets were used.

Second, we manipulated the extent of item-specific error per facet (latent factor) across three levels: low, mediocre, and high. In the condition with low item-specific error per facet, the error variances were low in all items measuring each facet (i.e., normally distributed errors with *N*[0, 0.5] for all items of each factor). In the condition with mediocre item-specific error per facet, the error variances were low for one half of the items of each facet (i.e., normally distributed errors with *N*[0, 0.5]) and high for the other half of the items of each facet (i.e., normally distributed errors with *N*[0, 1.5] ). In the condition with high item-specific error per facet, the error variances were high in all items measuring each facet (i.e., normally distributed errors with *N*[0, 1.5] for all items of each factor). An unstandardized factor loading of 1 corresponded to a standardized factor loading of .77 if the item-specific error variance was set to 0.5 and corresponded to a standardized factor loading of .40 if the item-specific error variance was set to 1.5. These levels of item-specific error were chosen because they reflect typical levels of measurement quality that may be achieved in confirmatory factor models: In the ideal case (i.e., low item-specific error condition), the latent factor explains the majority of variance in each of the construct items, which is given when the standardized loading is above .7 (Kline, 2016, p. 301). In applied settings, however, the more common case is (i.e., mediocre item-specific error condition) that only some of the items have standardized loadings above .7. In the worst case (i.e., high item-specific error condition), all item loadings are only around .4, which has also been suggested as the minimum value for a factor loading to be considered meaningful (Brown, 2015, p. 115). With an increasing extent of item-specific error per facet, the error in the scores of the scale half pairings and the resulting PR should increase as well. Thus, we expected PR and RPR to be more accurate indicators of careless responding when the extent of item-specific error per facet was low than when it was mediocre or even high.

Third, we examined the performance of PR and RPR across two patterns or types of careless responding: random uniform and invariant. These two careless response patterns were chosen because they represented two commonly applied strategies that survey participants may use when completing a questionnaire carelessly (Ward & Meade, 2023, pp. 581–582), and they have also been used in recent simulation studies on careless responding (Hong et al., 2020; Wind & Wang, 2023). In the random uniform careless responding pattern, all six response options (1 to 6) in the simulated response protocols had an equal probability of occurrence. In the case of the invariant careless responding pattern, however, only the two response options “4” and “5” were randomly simulated for the careless response protocols with an equal probability of occurrence. To obtain these invariant random response options, we drew from a normal distribution with *N*(4.5, 0.2) and rounded the drawn values to the next integer. Random uniform careless response patterns are therefore characterized by a large intra-facet response variance and invariant careless response patterns by a relatively small one. In turn, random uniform careless response patterns should therefore go along with far more response inconsistencies than the invariant careless response patterns, especially as all items were unidirectionally (i.e., positively) keyed in our simulation study. Thus, we expected PR and RPR to be more accurate in detecting random uniform than in detecting invariant careless response patterns.

Fourth, we manipulated the severity of careless responding in the careless response protocols across two levels: full and partial careless responding. These two levels of severity were chosen because they represented two types of careless respondents that studies on careless responding (Bowling et al., 2021; Meade & Craig, 2012; Ward & Meade, 2023; Yu & Cheng, 2019) have typically reported—participants that are unmotivated from the beginning, who complete the whole questionnaire carelessly and participants that lose the motivation during the completion of the survey, who answer the items carelessly after the “change-point” (Bowling et al., 2021; Yu & Cheng, 2019). In the full careless responding condition, all item responses of every careless response protocol were replaced with simulated careless responses. In the partial careless responding condition, 50% of the item responses of the response protocol were randomly selected and replaced with simulated careless responses.^4^ Generally, response protocols in which all item responses were simulated to be given carelessly tend to be more easily spotted than protocols in which only a partial number of item responses were simulated to be given carelessly (Hong et al., 2020; Meade & Craig, 2012). We expected PR and RPR to be more accurate in detecting full than in detecting partial careless responding.

### Summary of hypotheses

In sum, we expected PR and RPR to be more accurate when the conditions were favorable for them—conditions in which their computation is less affected by error (i.e., sampling or measurement error) and conditions in which the careless response patterns are easier to spot: when many facets/scale half pairings (e.g., 30) can be used for computation; when the extent of item-specific error per facet is low; when a random uniform careless response pattern should be detected; and when a careless response protocol should be detected in which all item responses are simulated to be given carelessly.

Moreover, because RPR is less affected by fluctuations than the conventional PR measure, which is based on only a single set of scale half parings (e.g., Curran, 2016), we expected RPR to outperform PR to a larger degree in conditions in which the fluctuations of the individual sets of scale half parings and their PR values are large: when fewer scale half pairings (e.g., only five) are used for computation; when the extent of item-specific error per facet is large; when careless response patterns should be detected that are harder to spot (e.g., invariant and partial careless response patterns).

### Fixed parameters

In all of our simulation conditions, the following parameters were held constant (see Table 4). First, we always drew samples with 300 observations and randomly defined 30% of them as careless respondents (whose careful response protocols were later on replaced with the careless response patterns). We held the sample size and the percentage of careless respondents in the sample constant across conditions, because we did not expect these two parameters to have an effect on the performance of the PR measures, which have the (in)consistency of the individual response protocols and not deviations from a normative response pattern as their primary focus (Goldammer et al., 2020; 2024). A sample size of 300 and a percentage of 30% careless respondents in the sample was chosen because comparable values had been used in previous simulation studies (Hong et al., 2020; Wind & Wang, 2023).

Second, we always drew the samples from a multivariate normal facet (latent factor) correlation matrix in which the facet correlations were set to .3 and the facet means and standard deviations were set to 3.5 and 0.7. As for sample size and percentage of careless respondents in the sample, the facet correlation was held constant across conditions, because we did not expect this parameter to have a large impact on the performance of the PR measures. Compared to the between-facet correlation, we considered the within-facet correlation (i.e., correlations among items in each facet) as more important for the performance of the PR measures, which is why we manipulated the extent of item-specific error per facet instead. A facet correlation of .3 was chosen because it approximated facet and domain correlations reported in validation studies (e.g., Soto & John, 2017) reasonably well, and by drawing samples from a multivariate normal facet correlation matrix, we followed previous simulation studies that used similar parameter settings for the data-generating process (Hong et al., 2020; Wind & Wang, 2023).

Third, for each of these drawn of facets (latent factors), we always generated four positively keyed items, and for each of these four items the factor loading was set to 1. The resulting continuous item scores were then rounded to the next integer, which allowed us to obtain a categorical response format in which the item values ranged from 1 to 6. If rounded item values fell below 1, they were recoded to 1, and if rounded item values were greater than 6, they were recoded to 6. We chose to use four items per facet and a categorical six-point response format because we considered these settings to be a reasonable approximation of the typical questionnaire format used in psychological assessments. By generating positively keyed items, we followed previous simulation studies that used similar parameter settings for the data-generating process (Hong et al., 2020; Wind & Wang, 2023). Lastly, the factor loadings for the items were held constant across conditions, because we already manipulated the measurement precision in the facets by using increasing numbers of less reliable items per facet.

### Measures of personal reliability

In addition to the conventional PR measure (i.e., even–odd consistency), we calculated three RPR versions that were based on a different number of independently drawn resamples (i.e., 25, 50, 100). Calculating these three RPR versions allowed us to examine their relative performance and thus to gain insights on the number of RPR resamples that is necessary to achieve the expected gain in detection accuracy over the conventional PR measure. The values of the conventional PR as well as those of the three RPR versions were all corrected with the Spearman–Brown prophesy formula.

### Outcome measures and analytical procedure

We used two classification accuracy measures as outcomes in our simulation study: the area under the receiver operating characteristic curve (AUC) and the sensitivity at a false-positive rate of 5%. The AUC statistic varies between 0 and 1 and can be interpreted as the probability that a randomly chosen carelessly responding individual has a higher score on a careless response index than a randomly chosen carefully responding individual (Pepe, 2000). Thus, an index can be considered as effective in detecting carelessly responding participants if its AUC is significantly larger than 0.5 (Lasko et al., 2005; Streiner & Cairney, 2007). In contrast, a slightly different picture is obtained by sensitivity at a false-positive rate of 5%. This measure indicates the percentage of careless response protocols that can be detected with a given index if we accept that 5% of the normal response protocols are falsely classified as being careless (e.g., Huang et al., 2012, p. 106).

We obtained these two outcome measures by running nonparametric receiver operating characteristic (ROC) regression models (using the Stata command *rocreg* with tie correction and bootstrapping) in which the PR measures were entered as independent variables and the protocol classification (careful vs. careless) as a dependent variable. The AUCs and sensitivities were then used in two different ways to examine the performance of PR and the three RPR versions. For one, we calculated averaged AUCs and sensitivities across the 100 replications of each condition. For another, we determined the effect size of our manipulated parameters on the AUCs and sensitivities. We therefore ran analysis of variance (ANOVA) models for each of the four PR measures and the two outcome measures, in which the manipulated parameters were entered as categorical independent variables and the AUC or the sensitivity as a dependent variable.

### Results

The averaged condition-specific AUC values are displayed in Table 5, the averaged condition-specific sensitivity values in Table 6, and the averaged condition-specific cutoff values at a false-positive rate of 5% in Table 7. In addition, Table 8 shows the effect sizes of the manipulated factors and their interactions on the AUCs and sensitivities of the conventional PR measure and the three RPR versions. When analyzing the simulation data, we considered effects as potentially meaningful only if they were significant at *p* < .001 and had an effect size of η^2^ > .01.

**Table 5:** Area under the curve for the conventional personal reliability and three resampled personal reliability versions across simulation conditions

Error per facet	Type of CR	PR	RPR25	RPR50	RPR100
Full careless responding					
5 Facets in survey					
Low	Invariant	0.839 (0.024)	0.906 (0.018)	0.907 (0.018)	0.908 (0.018)
Low	Uniform	0.844 (0.024)	0.905 (0.017)	0.906 (0.017)	0.907 (0.017)
Mediocre	Invariant	0.655 (0.031)	0.733 (0.030)	0.734 (0.030)	0.734 (0.030)
Mediocre	Uniform	0.662 (0.036)	0.729 (0.033)	0.730 (0.033)	0.730 (0.033)
High	Invariant	0.582 (0.034)	0.652 (0.034)	0.654 (0.033)	0.653 (0.034)
High	Uniform	0.584 (0.033)	0.645 (0.031)	0.645 (0.030)	0.646 (0.031)
15 Facets in survey					
Low	Invariant	0.979 (0.008)	0.994 (0.003)	0.994 (0.003)	0.994 (0.003)
Low	Uniform	0.979 (0.008)	0.994 (0.003)	0.994 (0.003)	0.994 (0.003)
Mediocre	Invariant	0.830 (0.026)	0.882 (0.018)	0.883 (0.018)	0.884 (0.019)
Mediocre	Uniform	0.828 (0.024)	0.880 (0.020)	0.882 (0.021)	0.882 (0.021)
High	Invariant	0.712 (0.033)	0.756 (0.033)	0.757 (0.032)	0.757 (0.032)
High	Uniform	0.716 (0.030)	0.761 (0.025)	0.762 (0.025)	0.763 (0.024)
30 Facets in survey					
Low	Invariant	0.999 (0.001)	1 (0)	1 (0)	1 (0)
Low	Uniform	0.999 (0.001)	1 (0)	1 (0)	1 (0)
Mediocre	Invariant	0.916 (0.019)	0.956 (0.012)	0.956 (0.012)	0.957 (0.012)
Mediocre	Uniform	0.917 (0.015)	0.954 (0.011)	0.955 (0.011)	0.956 (0.011)
High	Invariant	0.799 (0.028)	0.845 (0.026)	0.846 (0.025)	0.846 (0.025)
High	Uniform	0.800 (0.030)	0.848 (0.025)	0.849 (0.026)	0.849 (0.025)
Partial careless responding					
5 Facets in survey					
Low	Invariant	0.765 (0.032)	0.839 (0.022)	0.840 (0.022)	0.841 (0.022)
Low	Uniform	0.819 (0.024)	0.880 (0.020)	0.881 (0.019)	0.882 (0.019)
Mediocre	Invariant	0.610 (0.039)	0.665 (0.036)	0.666 (0.035)	0.666 (0.036)
Mediocre	Uniform	0.632 (0.031)	0.693 (0.032)	0.694 (0.032)	0.694 (0.032)
High	Invariant	0.550 (0.037)	0.592 (0.036)	0.594 (0.037)	0.594 (0.037)
High	Uniform	0.564 (0.038)	0.614 (0.031)	0.614 (0.034)	0.614 (0.034)
15 Facets in survey					
Low	Invariant	0.930 (0.016)	0.966 (0.009)	0.967 (0.010)	0.967 (0.009)
Low	Uniform	0.967 (0.009)	0.987 (0.006)	0.988 (0.006)	0.988 (0.006)
Mediocre	Invariant	0.731 (0.031)	0.784 (0.028)	0.785 (0.028)	0.786 (0.027)
Mediocre	Uniform	0.774 (0.029)	0.827 (0.025)	0.829 (0.025)	0.829 (0.025)
High	Invariant	0.647 (0.038)	0.679 (0.030)	0.681 (0.030)	0.681 (0.030)
High	Uniform	0.671 (0.031)	0.709 (0.032)	0.711 (0.032)	0.711 (0.031)
30 Facets in survey					
Low	Invariant	0.983 (0.007)	0.995 (0.003)	0.995 (0.003)	0.995 (0.003)
Low	Uniform	0.996 (0.002)	0.999 (0.001)	0.999 (0.001)	0.9995 (0.001)
Mediocre	Invariant	0.813 (0.030)	0.866 (0.025)	0.867 (0.024)	0.868 (0.024)
Mediocre	Uniform	0.872 (0.020)	0.920 (0.014)	0.920 (0.015)	0.921 (0.015)
High	Invariant	0.693 (0.033)	0.735 (0.029)	0.735 (0.029)	0.736 (0.029)
High	Uniform	0.742 (0.028)	0.787 (0.025)	0.788 (0.026)	0.788 (0.026)

*Note.* Numbers presented are means of areas under the receiver operating characteristic curves across the 100 replications per condition (*SD*s in paratheses). Type of CR = type of careless responding; PR = conventional personal reliability (i.e., even–odd consistency); RPR25 = mean-based resampled personal reliability with 25 sets of scale half pairings; RPR50 = mean-based resampled personal reliability with 50 sets of scale half pairings; RPR100 = mean-based resampled personal reliability with 100 sets of scale half pairings; Low = low item error per facet with normally distributed errors with *N*(0, 0.5) for all items of each factor; Mediocre = mediocre item error per facet with normally distributed errors with *N*(0, 0.5) for one half of the items of each factor and normally distributed errors with *N*(0, 1.5) for the other half of the items of each factor; High = high item error per facet with normally distributed errors with *N*(0, 1.5) for all items of each factor; Invariant = invariant careless responding (i.e., only the two response options “4” and “5” were randomly simulated for the careless response protocols with an equal probability of occurrence); Uniform = uniform random careless responding (i.e., all six response options (1 to 6) in the simulated response protocols had an equal probability of occurrence); Full careless responding = all item responses of the response protocol were replaced with simulated careless responses; Partial careless responding = 50% of the item responses of the response protocol were randomly selected and replaced with simulated careless responses

**Table 6:** Sensitivity at a false-positive rate of 5% for the conventional personal reliability and three resampled personal reliability versions across simulation conditions

Error per facet	Type of CR	PR	RPR25	RPR50	RPR100
Full careless responding					
5 Facets in survey					
Low	Invariant	0.413 (0.074)	0.513 (0.087)	0.521 (0.089)	0.524 (0.084)
Low	Uniform	0.435 (0.087)	0.494 (0.093)	0.500 (0.086)	0.500 (0.088)
Mediocre	Invariant	^a^	0.183 (0.044)	0.181 (0.045)	0.178 (0.043)
Mediocre	Uniform	^a^	0.152 (0.055)	0.155 (0.056)	0.153 (0.059)
High	Invariant	^a^	0.176 (0.041)	0.178 (0.038)	0.178 (0.038)
High	Uniform	^a^	0.103 (0.044)	0.101 (0.043)	0.105 (0.040)
15 Facets in survey					
Low	Invariant	0.896 (0.046)	0.977 (0.022)	0.979 (0.019)	0.980 (0.021)
Low	Uniform	0.901 (0.042)	0.979 (0.015)	0.977 (0.017)	0.978 (0.015)
Mediocre	Invariant	0.386 (0.069)	0.475 (0.073)	0.481 (0.078)	0.474 (0.080)
Mediocre	Uniform	0.384 (0.070)	0.473 (0.089)	0.472 (0.092)	0.475 (0.092)
High	Invariant	0.201 (0.066)	0.223 (0.074)	0.229 (0.075)	0.227 (0.074)
High	Uniform	0.204 (0.060)	0.238 (0.061)	0.242 (0.065)	0.244 (0.064)
30 Facets in survey					
Low	Invariant	0.996 (0.007)	0.9997 (0.002)	0.9998 (0.002)	0.9999 (0.001)
Low	Uniform	0.994 (0.007)	0.9999 (0.001)	0.9999 (0.001)	0.9999 (0.001)
Mediocre	Invariant	0.620 (0.077)	0.759 (0.075)	0.764 (0.074)	0.766 (0.070)
Mediocre	Uniform	0.629 (0.069)	0.758 (0.062)	0.761 (0.065)	0.764 (0.065)
High	Invariant	0.321 (0.071)	0.387 (0.082)	0.390 (0.085)	0.389 (0.085)
High	Uniform	0.322 (0.081)	0.395 (0.092)	0.404 (0.090)	0.403 (0.094)
Partial careless responding					
5 Facets in survey					
Low	Invariant	0.287 (0.063)	0.321 (0.077)	0.328 (0.077)	0.329 (0.077)
Low	Uniform	0.368 (0.071)	0.408 (0.088)	0.403 (0.089)	0.400 (0.092)
Mediocre	Invariant	^a^	0.113 (0.049)	0.111 (0.039)	0.112 (0.043)
Mediocre	Uniform	^a^	0.133 (0.050)	0.131 (0.056)	0.134 (0.053)
High	Invariant	^a^	0.093 (0.040)	0.093 (0.040)	0.093 (0.041)
High	Uniform	^a^	0.088 (0.035)	0.086 (0.038)	0.088 (0.034)
15 Facets in survey					
Low	Invariant	0.691 (0.073)	0.819 (0.063)	0.822 (0.063)	0.823 (0.060)
Low	Uniform	0.834 (0.055)	0.935 (0.040)	0.938 (0.037)	0.940 (0.036)
Mediocre	Invariant	0.228 (0.064)	0.272 (0.067)	0.275 (0.069)	0.276 (0.072)
Mediocre	Uniform	0.278 (0.066)	0.342 (0.077)	0.352 (0.077)	0.349 (0.073)
High	Invariant	0.132 (0.048)	0.147 (0.049)	0.143 (0.048)	0.147 (0.049)
High	Uniform	0.159 (0.054)	0.175 (0.062)	0.178 (0.064)	0.175 (0.065)
30 Facets in survey					
Low	Invariant	0.917 (0.034)	0.975 (0.017)	0.975 (0.017)	0.977 (0.017)
Low	Uniform	0.985 (0.014)	0.999 (0.003)	0.999 (0.003)	0.999 (0.002)
Mediocre	Invariant	0.353 (0.072)	0.445 (0.079)	0.449 (0.084)	0.453 (0.088)
Mediocre	Uniform	0.481 (0.079)	0.607 (0.077)	0.611 (0.081)	0.610 (0.080)
High	Invariant	0.190 (0.058)	0.214 (0.064)	0.214 (0.065)	0.213 (0.063)
High	Uniform	0.237 (0.060)	0.296 (0.069)	0.295 (0.069)	0.300 (0.071)

*Note.* Numbers presented are means of the sensitivities at a false-positive rate of 5% across the 100 replications per condition (*SD*s in paratheses). Type of CR = type of careless responding; PR = conventional personal reliability (i.e., even–odd consistency); RPR25 = mean-based resampled personal reliability with 25 sets of scale half pairings; RPR50 = mean-based resampled personal reliability with 50 sets of scale half pairings; RPR100 = mean-based resampled personal reliability with 100 sets of scale half pairings; Low = low item error per facet with normally distributed errors with *N*(0, 0.5) for all items of each factor; Mediocre = mediocre item error per facet with normally distributed errors with *N*(0, 0.5) for one half of the items of each factor and normally distributed errors with *N*(0, 1.5) for the other half of the items of each factor; High = high item error per facet with normally distributed errors with *N*(0, 1.5) for all items of each factor; Invariant = invariant careless responding (i.e., only the two response options “4” and “5” were randomly simulated for the careless response protocols with an equal probability of occurrence); Uniform = uniform random careless responding (i.e., all six response options (1 to 6) in the simulated response protocols had an equal probability of occurrence); Full careless responding = all item responses of the response protocol were replaced with simulated careless responses; Partial careless responding = 50% of the item responses of the response protocol were randomly selected and replaced with simulated careless responses.

^a^ Sensitivities could not be computed because of missing values in the matrix (i.e., nonzero sensitivities could only be calculated for false-positive rates that were higher than the 5% level that we aimed for)

**Table 7:** Cutoff values at a false-positive rate of 5% for the conventional personal reliability and three resampled personal reliability versions across simulation conditions

Error per facet	Type of CR	PR	RPR25	RPR50	RPR100
Full careless responding					
5 Facets in survey					
Low	Invariant	–0.257 (0.249)	–0.130 (0.157)	–0.114 (0.154)	–0.115 (0.150)
Low	Uniform	–0.310 (0.246)	–0.140 (0.157)	–0.135 (0.147)	–0.127 (0.145)
Mediocre	Invariant	–1 (0)	–0.802 (0.068)	–0.792 (0.064)	–0.794 (0.064)
Mediocre	Uniform	–1 (0)	–0.783 (0.072)	–0.772 (0.069)	–0.770 (0.075)
High	Invariant	–1 (0)	–0.869 (0.047)	–0.863 (0.049)	–0.859 (0.048)
High	Uniform	–1 (0)	–0.868 (0.047)	–0.860 (0.047)	–0.854 (0.047)
15 Facets in survey					
Low	Invariant	0.515 (0.047)	0.565 (0.035)	0.566 (0.035)	0.566 (0.034)
Low	Uniform	0.518 (0.043)	0.563 (0.034)	0.565 (0.034)	0.564 (0.034)
Mediocre	Invariant	–0.200 (0.095)	–0.086 (0.077)	–0.078 (0.074)	–0.082 (0.073)
Mediocre	Uniform	–0.209 (0.102)	–0.089 (0.079)	–0.089 (0.081)	–0.085 (0.081)
High	Invariant	–0.647 (0.140)	–0.457 (0.091)	–0.451 (0.092)	–0.450 (0.090)
High	Uniform	–0.640 (0.144)	–0.435 (0.089)	–0.436 (0.090)	–0.428 (0.093)
30 Facets in survey					
Low	Invariant	0.638 (0.020)	0.667 (0.017)	0.667 (0.016)	0.667 (0.017)
Low	Uniform	0.639 (0.023)	0.664 (0.017)	0.666 (0.018)	0.666 (0.018)
Mediocre	Invariant	0.095 (0.048)	0.173 (0.040)	0.175 (0.040)	0.177 (0.040)
Mediocre	Uniform	0.106 (0.051)	0.177 (0.039)	0.176 (0.039)	0.179 (0.038)
High	Invariant	–0.203 (0.073)	–0.115 (0.058)	–0.111 (0.054)	–0.112 (0.055)
High	Uniform	–0.208 (0.077)	–0.117 (0.061)	–0.111 (0.058)	–0.110 (0.062)
Partial careless responding					
5 Facets in survey					
Low	Invariant	–0.324 (0.291)	–0.132 (0.152)	–0.126 (0.149)	–0.120 (0.143)
Low	Uniform	–0.323 (0.274)	–0.158 (0.157)	–0.162 (0.158)	–0.164 (0.170)
Mediocre	Invariant	–1 (0)	–0.784 (0.080)	–0.778 (0.076)	–0.773 (0.078)
Mediocre	Uniform	–1 (0)	–0.780 (0.076)	–0.773 (0.082)	–0.770 (0.078)
High	Invariant	–1 (0)	–0.865 (0.052)	–0.853 (0.056)	–0.853 (0.054)
High	Uniform	–1 (0)	–0.870 (0.044)	–0.862 (0.044)	–0.857 (0.042)
15 Facets in survey					
Low	Invariant	0.522 (0.045)	0.565 (0.038)	0.564 (0.037)	0.565 (0.037)
Low	Uniform	0.514 (0.039)	0.562 (0.037)	0.565 (0.036)	0.566 (0.035)
Mediocre	Invariant	–0.215 (0.101)	–0.084 (0.086)	–0.082 (0.078)	–0.080 (0.082)
Mediocre	Uniform	–0.227 (0.101)	–0.103 (0.078)	–0.091 (0.076)	–0.093 (0.073)
High	Invariant	–0.627 (0.127)	–0.438 (0.090)	–0.441 (0.088)	–0.435 (0.090)
High	Uniform	–0.656 (0.158)	–0.461 (0.098)	–0.454 (0.090)	–0.451 (0.094)
30 Facets in survey					
Low	Invariant	0.637 (0.021)	0.665 (0.016)	0.665 (0.017)	0.666 (0.018)
Low	Uniform	0.639 (0.018)	0.667 (0.016)	0.667 (0.015)	0.668 (0.015)
Mediocre	Invariant	0.095 (0.051)	0.170 (0.044)	0.173 (0.044)	0.175 (0.045)
Mediocre	Uniform	0.105 (0.054)	0.175 (0.047)	0.176 (0.046)	0.176 (0.047)
High	Invariant	–0.208 (0.081)	–0.114 (0.061)	–0.112 (0.064)	–0.111 (0.060)
High	Uniform	–0.195 (0.065)	–0.097 (0.055)	–0.098 (0.051)	–0.095 (0.054)

*Note.* Numbers presented are means of the cutoff values at a false-positive rate of 5% across the 100 replications per condition (*SD*s in paratheses). Type of CR = type of careless responding; PR = conventional personal reliability (i.e., even–odd consistency); RPR25 = mean-based resampled personal reliability with 25 sets of scale half pairings; RPR50 = mean-based resampled personal reliability with 50 sets of scale half pairings; RPR100 = mean-based resampled personal reliability with 100 sets of scale half pairings; Low = low item error per facet with normally distributed errors with *N*(0, 0.5) for all items of each factor; Mediocre = mediocre item error per facet with normally distributed errors with *N*(0, 0.5) for one half of the items of each factor and normally distributed errors with *N*(0, 1.5) for the other half of the items of each factor; High = high item error per facet with normally distributed errors with *N*(0, 1.5) for all items of each factor; Invariant = invariant careless responding (i.e., only the two response options “4” and “5” were randomly simulated for the careless response protocols with an equal probability of occurrence); Uniform = uniform random careless responding (i.e., all six response options (1 to 6) in the simulated response protocols had an equal probability of occurrence); Full careless responding = all item responses of the response protocol were replaced with simulated careless responses; Partial careless responding = 50% of the item responses of the response protocol were randomly selected and replaced with simulated careless responses

**Table 8:** Effect size (η^2^) of manipulated factors on the detection accuracy of conventional personal reliability and three resampled personal reliability versions

	Area under the curve				Sensitivity at a false-positive rate of 5%			
Source of effect	PR	RPR25	RPR50	RPR100	PR	RPR25	RPR50	RPR100
Main effects								
Error	0.939	0.945	0.945	0.944	0.948	0.938	0.937	0.937
Facets	0.909	0.902	0.902	0.901	0.890	0.890	0.888	0.888
Severity	0.457	0.533	0.533	0.531	0.432	0.425	0.427	0.424
Type of CR	0.107	0.102	0.103	0.102	0.088	0.045	0.043	0.044
Two-way interactions								
Error*Facets	0.198	0.381	0.386	0.387	0.099	0.632	0.620	0.623
Error*Severity	0.064	0.154	0.153	0.153	0.081	0.056	0.050	0.048
Error*Type of CR	0.002^b^	0.006	0.006	0.006	0.012	0.010	0.009	0.008
Facets*Severity	0.022	0.002^b^	0.001^b^	0.001^b^	0.002^b^	0.028	0.025	0.025
Facets*Type of CR	0.001^b^	0.002^b^	0.003^b^	0.003^b^	0.0003^b^	0.030	0.033	0.033
Severity*Type of CR	0.087	0.113	0.112	0.111	0.061	0.087	0.088	0.081
Three-way interactions								
Error*Facets*Severity	0.098	0.093	0.095	0.093	0.071	0.165	0.165	0.171
Error*Facets*Type of CR	0.019	0.021	0.022	0.022	0.017	0.031	0.028	0.026
Error*Severity*Type of CR	0.002^b^	0.009	0.008	0.008	0.012	0.006	0.006	0.005
Facets*Severity*Type of CR	0.003^b^	0.001^b^	0.001^b^	0.001^b^	0.003^b^	0.001^b^	0.001^b^	0.001^b^
Four-way interactions								
Error*Facets*Severity*Type of CR	0.020	0.016	0.016	0.016	0.011	0.025	0.024	0.024

*Note.* PR = Conventional personal reliability (i.e., even–odd consistency); RPR25 = mean-based resampled personal reliability that is based on 25 sets of scale half pairings; RPR50 = mean-based resampled personal reliability that is based on 50 sets of scale half pairings; RPR100 = mean-based resampled personal reliability that is based on 100 sets of scale half pairings; Error = error per facet (i.e., low, mediocre, high item error per facet); Facets = facet in survey used calculation personal reliability measures (i.e., 5, 15, 30); Severity = severity of careless responding in response protocols (i.e., full, partial); Type of CR = type of careless responding (i.e., invariant, uniform random). All effects were significant at *p* < .001, except for those marked with superscript ^b^

^b^ *p* > .001

#### Impact of manipulated factors on the detection accuracy of the PR measures

As expected, the conventional PR measure and the three RPR versions were more accurate in detecting careless responding in conditions in which their computation was less affected by error (i.e., conditions in which more facets and facets with lower item-specific error were used) and in conditions in which the careless response patterns were easier to spot (i.e., conditions in which full careless responding and uniform random careless response patterns should be detected).

With η^2^ values of .89 and above, the error per facet and the number of facets had the strongest main effects on the AUC and sensitivity of the four PR measures (see Table 8). Thus, higher AUC and sensitivity values could be obtained for the conventional PR measure and the RPR versions if more facets and facets with a low item-specific error were for used for computation. With η^2^ values of .42 and above, the severity of the careless responding in the response protocols (i.e., full vs. partial careless responding) also had a strong main effect on the AUC and sensitivity of the four PR measures (see Table 8). Thus, higher AUC and sensitivity values could be obtained for the conventional PR measure and the three RPR versions if full rather than partial careless responding should be detected. Compared to the other manipulated factors, the smallest main effect, with η^2^ values of .04 (see Table 8), was observed for type of careless responding (i.e., invariant vs. uniform random), indicating that invariant careless responding was harder to detect than uniform random responding.

**Fig. 1:**
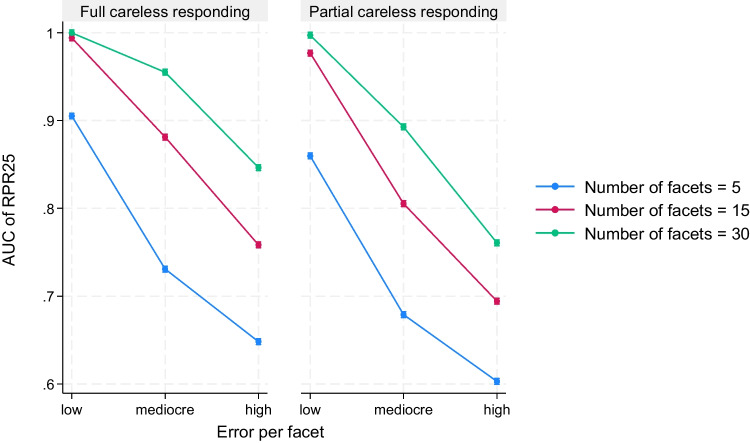
Three-way interaction between error per facet, number of facets, and severity of careless responding when predicting AUC of RPR25. *Note*. Results were based on 3600 replications, *F*(4, 3564) = 90.98, *p* < .001, η^2^ = .09. AUC of RPR25 = area under the receiver operating characteristic curve for the mean-based resampled personal reliability with 25 sets of scale half pairings (higher values indicate a higher probability of detecting careless responding); Low = low item error per facet with normally distributed errors with *N*(0, 0.5) for all items of each factor; Mediocre = mediocre item error per facet with normally distributed errors with *N*(0, 0.5) for one half of the items of each factor and normally distributed errors with *N*(0, 1.5) for the other half of the items of each factor; High = high item error per facet with normally distributed errors with *N*(0, 1.5) for all items of each factor; Full careless responding = all item responses of the response protocol are simulated to be given carelessly; Partial careless responding = 50% of the item responses of the response protocol were randomly selected and replaced with simulated careless responses

In addition to these main effects, there were also some interesting two-way and three-way interaction effects between the manipulated factors (see Table 8). For instance, the error per facet interacted with the number of facets (with η^2^ values that ranged from .10 to .63) and the severity of careless responding in the response protocols (with η^2^ values that ranged from .05 to .15), and these three factors were all part of a three-way interaction (with η^2^ values that ranged from .07 to .17). The three-way interaction indicated that the decrease in AUC and sensitivity that occurred when shifting from low to high error per facet was more pronounced when five instead of 30 facets were used, especially when partial instead of full careless responding should be detected. In Fig. 1, this three-way interaction is displayed for the case when the AUC of the RPR with 25 resamples (RPR25) is used as outcome. However, this interaction pattern was comparable to the pattern that we observed when the sensitivity of RPR25 was used and when AUC or sensitivity values of other PR measures were used as outcome.

**Fig. 2:**
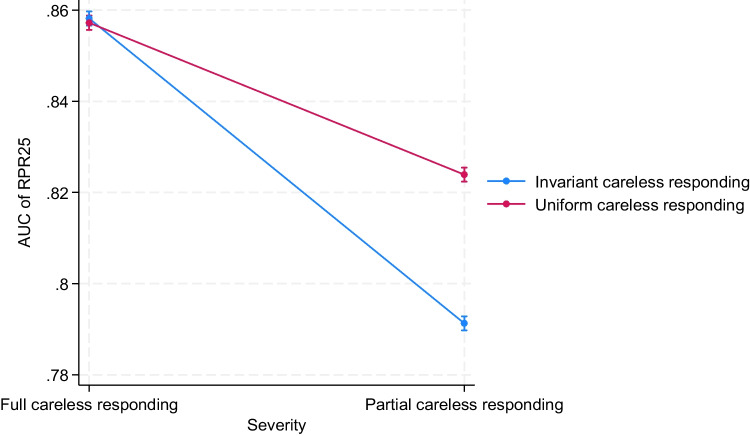
Two-way interaction between severity of careless responding and type of careless responding when predicting AUC of RPR25. *Note*. Results were based on 3600 replications, *F*(4, 3564) = 455.77, *p* < .001, η^2^ = .11. AUC of RPR25 = area under the receiver operating characteristic curve for the mean-based resampled personal reliability with 25 sets of scale half pairings (higher values indicate a higher probability of detecting careless responding); Full careless responding = all item responses of the response protocol were simulated to be given carelessly; Partial careless responding = 50% of the item responses of the response protocol were randomly selected and replaced with simulated careless responses; Invariant careless responding = only the two response options “4” and “5” were randomly simulated for the careless response protocols with an equal probability of occurrence; Uniform careless responding = all six response options (1 to 6) in the simulated response protocols had an equal probability of occurrence

For another, the severity of careless responding in the response protocols interacted with the type of careless responding (with η^2^ values that ranged from .06 to .11). This two-way interaction indicated the following: When all item responses of the response protocols were simulated to be given carelessly, invariant and uniform random careless responding could be detected equally well. However, when only 50% of the items of the response protocols were randomly selected and replaced with simulated careless responses, invariant careless responding was harder to detect than uniform random careless responding. In Fig. 2, this two-way interaction is displayed for the case when the AUC of the RPR with 25 resamples (RPR25) is used as outcome. However, this interaction pattern was comparable to the pattern that we observed when the sensitivity of RPR25 was used and when AUC or sensitivity values of other PR measures were used as outcome.

#### Comparison of PR and RPR across simulation conditions

To examine whether the conventional PR measure and the three RPR versions performed differently across the simulation conditions, we inspected the indices shown in Tables 5 and 6 and additionally ran ANOVA models with a data set in long format in which the four PR measures were represented as additional predictor (with four levels) and their replication-specific AUC and sensitivity values as four separate row entries listed under a single AUC and sensitivity column.

With an η^2^ value of .39 in the case of AUC and an η^2^ value of .14 in the case of sensitivity, the PR predictor had a substantial impact on the two outcomes measures. In line with our expectation, this main effect indicated that the three RPR measures generally performed better in detecting careless responding than the conventional PR measure.

Across all conditions, AUC improved from .789 to .833–.834, and sensitivity from .494 to .558–.561 when using an RPR version instead of PR. When using an RPR version instead of the conventional PR measure, the largest gain in the AUC (+.078–.079) occurred if five facets with mediocre item-specific error were used and invariant full careless responding should be detected, and the smallest gain in AUC (+.001) occurred if 30 facets with low item-specific error were used and uniform random full careless responding should be detected. Similarly, when using a RPR version instead of the conventional PR measure, the largest gain in sensitivity (+.178–.183) occurred if five facets with mediocre item-specific error were used and invariant full careless responding should be detected, and the smallest gain in sensitivity (+.004) occurred if 30 facets with low item-specific error were used and invariant full careless responding should be detected.

Bonferroni-corrected pairwise comparisons between the PR measures further revealed no significant differences in detection performance (i.e., AUC and sensitivity) between the three RPR versions. Thus, using 25 resamples for the RPR computation was sufficient to obtain the expected gain in detection accuracy over the conventional PR measure, and using more resamples (i.e., 50 or 100) was not associated with an additional improvement in AUC and sensitivity values.^5^

**Fig. 3:**
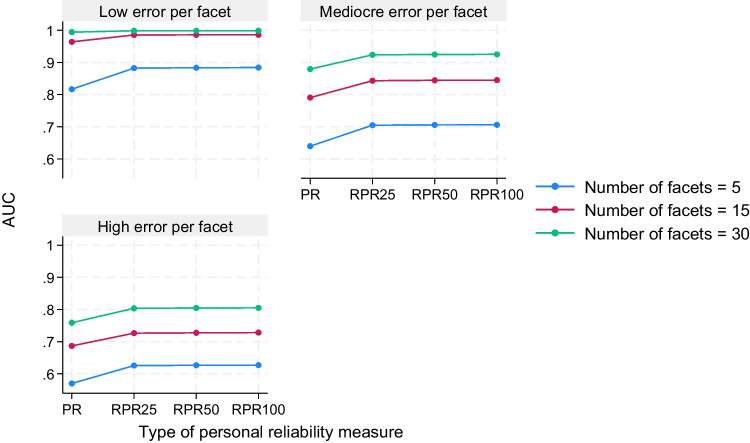
Three-way interaction between type of personal reliability measure, number of facets, and error per facet when predicting AUC. *Note*. The four personal reliability (PR) measures were calculated in each of the 3600 replications. To examine the interaction, a data set in long format was used in which the AUCs of the PR measures were listed as four separate rows under a single AUC column. Unadjusted test statistic is equal to *F*(12, 14,256) = 33.60, *p* < .001. Adjusted test statistic that is based on cluster-robust *SE* with replication id as cluster variable is equal to *F*(12, 3599) = 94.86, *p* < .001. The effect size for this interaction is equal to η^2^ = .03 (using ordinary least-squares estimation). PR = conventional personal reliability (i.e., even–odd consistency); AUC = area under the receiver operating characteristic curve (higher values indicate a higher probability of detecting careless responding); RPR25 = resampled personal reliability that is based on 25 sets of scale half pairings; RPR50 = resampled personal reliability that is based on 50 sets of scale half pairings; RPR100 = resampled personal reliability that is based on 100 sets of scale half pairings; Low error per facet = normally distributed errors with *N*(0, 0.5) for all items of each factor; Mediocre error per facet = normally distributed errors with *N*(0, 0.5) for one half of the items of each factor and normally distributed errors with *N*(0, 1.5) for the other half of the items of each factor; High error per facet = normally distributed errors with *N*(0, 1.5) for all items of each factor

In addition to these main effects, there was an interesting pattern of two-way and three-way interaction effects between the PR predictor and the other manipulated factors. The PR predictor interacted with number of facets (η^2^ = .05) and the error per facet (η^2^ = .03) when predicting AUC, and these three factors were all part of a three-way interaction when predicting AUC (η^2^ = .03) and sensitivity (η^2^ = .03). This three-way interaction indicated that the gain in detection accuracy that occurred when changing from PR to RPR25 (for instance) was more pronounced when five instead 30 facets were used, especially when the error per facet was low instead of high. In Fig. 3, this three-way interaction is displayed for the case when AUC was used as outcome. However, this interaction pattern was comparable to the one that we observed when sensitivity was used as outcome. Thus, our expectation that the RPR would outperform the PR to a larger degree in conditions in which the fluctuations in the individual sets of scale half parings and their PR values is large was at least supported for a specific set of simulation conditions.

## Reanalysis of real data

### Study setting and participants

For our real data example, we reanalyzed the data from Goldammer et al.’s (2020) experimental study on careless responding. That study sample consisted of 359 German-speaking, predominantly male (*n* = 357; 99.4%) military conscripts who were on average 20 years (*SD* = 1.14) old. The conscripts were nested in 12 platoons, of which each had on average 29.92 members (*SD* = 8.49). Each platoon was led by one platoon leader, and at the time the study took place, the conscripts had been led by their platoon leader for about 9 to 10 weeks (Goldammer et al., 2020). Every conscript received 10 Swiss francs for participation.

### Substantive study measures

The substantive questionnaire in Goldammer et al. (2020) contained six broad scales: three leader behavior measures (i.e., transformational, passive-avoidant, and authentic leadership), two relational correlates of leadership (leader–member exchange, organizational commitment), and one follower effectiveness criterion (organizational citizenship behavior).

Transformational leadership (TFL) was assessed with the German adaptation (Felfe, 2006) of the Multifactor Leadership Questionnaire (MLQ; Bass & Avolio, 1995). The German TFL scale included six facets, and each of these facets contained four items that were rated on a five-point Likert scale ranging from 1 = *never* to 5 = *frequently, almost always*. Passive-avoidant leadership (PAL) was assessed with the German adaptation of the two MLQ facets management-by-exception passive and laissez-faire (Bass & Avolio, 1995). Each of these two facets was measured with four items on a five-point Likert scale that ranged from 1 = *never* to 5 = *frequently, almost always*. Authentic leadership (AL) was assessed with the publisher’s (i.e., Mindgarden) German translation of the Authentic Leadership Questionnaire (Avolio et al., 2007). Of the four AL facets, the transparency facet was assessed by five items, the moral and self-awareness facets were each assessed by four items, and the balanced processing facet by three items. All AL items were rated on a five-point Likert scale that ranged from 1 = *never* to 5 = *frequently, almost always*. Leader–member exchange (LMX) was assessed with the German version (Paul & Schyns, 2002) of Liden and Maslyn’s (1998) multidimensional measure. LMX included four facets, and each facet was measured by three items that were rated on a five-point Likert scale that ranged from 1 = *does not apply at all* to 5 = *applies completely*.

Further, organizational commitment (OC) was assessed with the German adaptation (COBB; Felfe & Franke, 2012) of Meyer and Allen’s (1990) commitment measure. Of the three facets of OC, the affective and normative facet were each assessed by five items and the continuance facet by four items. All OC items were rated on a five-point Likert scale that ranged from 1 = *does not apply at all* to 5 = *applies completely*. The conscript’s organizational citizenship behavior (OCB) was assessed with the German adaptation (Staufenbiel & Hartz, 2000) of the OCB scale proposed by Podsakoff et al. (1990). OCB included four facets, and each facet was measured by five items that ranged from 1 = *does not apply at all* to 5 = *applies completely*. In sum, the questionnaire in Goldammer et al. (2020) included 23 facets that were measured by 94 items.

### Experimental conditions and survey arrangement

In Goldammer et al. (2020), participants were randomly assigned to one of three response conditions: careful responding (*n* = 121), random careless responding (*n* = 119), and opposite careless responding (*n* = 119). The participants in the careful responding condition were instructed to complete all items accurately and attentively. The participants in the random responding condition were instructed to select any response option they wanted on 50% of the items in each scale (i.e., TFL, PAL, AL, LMX, OC, OCB). The participants in the opposite responding condition were instructed to select the opposite of the response option that would have actually applied to them on 50% of the items in each scale.

The questionnaire in Goldammer et al. (2020) was arranged in six randomly ordered scale-specific blocks; each scale-specific block was further divided into two survey pages. On the first survey page of each block, a random selection of 50% of the items of each scale was displayed. For this item selection, all participants were instructed to respond carefully. The remaining 50% of the scale-specific items were displayed on the following second survey page of each block, for which the participants received the condition-specific response instructions.

### Measures of personal reliability

As we did for the simulation study, we calculated the conventional PR measure (i.e., even–odd consistency) and three RPR versions that were based on a different number of independently drawn resamples (i.e., 25, 50, 100).

### Outcome measures and analytical procedure

We used the AUC and the sensitivity at a false-positive rate of 5% as outcome measures for the real data analyses. We obtained these two outcome measures by running nonparametric ROC regression models (using the Stata command *rocreg* with tie correction and bootstrapping) in which the PR measures were entered as independent variables and the condition assignment (careful vs. careless) as a dependent variable. We calculated these two outcome measures for each of our four PR measures across three facet levels: 6, 16, and 23. For the first facet level (i.e., 6), only the six TFL facets and their 24 items were used for the computation of the four PR measures and their AUCs and sensitivities. For the second facet level (i.e., 16), the 16 facets of the TFL, PAL, AL, and LMX scales and their 60 items were used for the computation of the four PR measures and their AUCs and sensitivities. For the third facet level (i.e., 23), all 23 facets and their 94 items were used for the computation of the four PR measures and their AUCs and sensitivities. This procedure allowed us to examine the performance of the PR measures across facet levels that were comparable to those evaluated in our simulation study.

### Results

Table 9 shows the AUCs and sensitivities of the four PR measures for each of the three facet “conditions.” The omnibus test of equality indicated a significant inequality between the AUCs of the four PR measures in the 16-facet condition, χ^2^(3) = 12.44, *p* = .006, and the 23-facet condition, χ^2^(3) = 12.30, *p* = .006. In both of these facet conditions, Bonferroni-corrected pairwise post hoc comparisons further revealed significant differences between the conventional PR measure and each of the three RPR versions. In addition, the pairwise comparisons showed no significant differences in AUC between the three RPR versions in either of these facet conditions. Even though the Bonferroni-corrected critical χ^2^ value was not reached when testing the AUCs for equality in the six-facet condition, we observed the same pattern of AUC results as in the 16- and 23-facet conditions (i.e., comparable AUCs for the three RPR versions, which all tended to be higher than that of the conventional PR measure). In contrast to the omnibus test for AUC equality, none of the three omnibus tests for sensitivity equality reached the Bonferroni-corrected critical χ^2^ value of 11.74. Nevertheless, we observed a comparable pattern as in the AUC results (i.e., comparable sensitivities for the three RPR versions, which all tended to be higher than that of the conventional PR measure).

**Table 9:** Careless responding detection accuracy for the conventional personal reliability and three resampled personal reliability versions

Classification accuracy measures	PR	RPR25	RPR50	RPR100	χ^2^(3)
6 Facets					
AUC [95% CI]	.650 [.591, .710]	.687 [.627, .746]	.693 [.635, .752]	.684 [.624, .743]	8.88
SEN95	^a^	.092	.113	.105	0.44
16 Facets					
AUC [95% CI]	.933 [.905, .961]^a^	.955 [.934, .976]^b^	.956 [.935, .976]^b^	.959 [.940, .979]^b^	12.44^*^
SEN95	.744	.815	.786	.828	3.13
23 Facets					
AUC [95% CI]	.942 [.918, .966]^a^	.963 [.944, .981]^b^	.965 [.947, .983]^b^	.964 [.946, .982]^b^	12.30^*^
SEN95	.749	.845	.887	.870	3.88

*Note*. The total sample (*n* = 359) was used for computation. All personal reliability measures were inverted prior to the calculation of the classification accuracy measures. Tie-corrected nonparametric receiver operating characteristic regression models with 1000 bootstrap samples were used for the of computation of the classification accuracy measures. PR = conventional personal reliability (i.e., even–odd consistency); RPR25 = resampled personal reliability that is based on 25 sets of scale half pairings; RPR50 = resampled personal reliability that is based on 50 sets of scale half pairings; RPR100 = resampled personal reliability that is based on 100 sets of scale half pairings; AUC = area under the receiver operating characteristic curve; SEN95 = sensitivity at a specificity level of 95 % (i.e., at false-positive rate of 5%); χ^2^ = χ^2^ values were obtained by conducting Wald tests of parameter constraints (i.e., constraining parameters in each row to equality). A global inequality between estimates in each row was further examined with Bonferroni-corrected pairwise comparisons (i.e., α = .05/6, with the critical χ^2^(1) value of 6.96); Estimates with different subscripts turned out to be significantly different in the Bonferroni-corrected pairwise comparisons; 6 facets = 6 facets of the questionnaire were used for the computation of the conventional and resampled personal reliability measures; 16 facets = 16 facets of the questionnaire were used for the computation of the conventional and resampled personal reliability measures; 23 facets = 23 facets of the questionnaire were used for the computation of the conventional and resampled personal reliability measures

^*^ Larger than the critical χ^2^(3) value of 11.74 (i.e., α = .05/6), which indicates global inequality between AUCs or sensitivities in each row

^a^ The SEN95 value for PR could not be computed when only six facets were used; alternative ROC analyses showed that 10% was the lowest possible false-positive rate with a nonzero sensitivity that could be reached with PR when only six facets were used for computation

The real data analyses therefore allowed us to obtain two insights. First, the increase in detection accuracy when shifting from the conventional PR measure to the RPR25 (for instance) did not seem to substantially decrease when using more facets for the PR calculation (e.g., when using 16 instead of six facets). Instead, it seemed that using RPR went along with a constant gain of AUC across the three examined facet levels. This result was contrary to our expectation but partially in line with the simulation results in which the supposed interaction effect (i.e., using RPR is associated with a larger gain in detection accuracy when conditions are rough) also only occurred for specific sets of conditions. The second and more important result, however, was that the RPR measures were generally more accurate than the conventional PR measure. This finding illustrated that RPR may be of greater utility not only when detecting computer-generated careless response patterns but also when human-generated careless response patterns need to be detected.

## Discussion

The aim of our present research was to examine whether RPR really outperforms the conventional PR or even–odd consistency in detecting careless responding, and under what conditions the potential gain in detection accuracy is the most pronounced. We therefore conducted a simulation study in which we evaluated the relative performance of the conventional PR and three RPR versions in detecting simulated careless response protocols across 36 conditions. In a second study, we examined the performance of PR and the three RPR versions when detecting human-generated careless response protocols by reanalyzing the data from Goldammer et al.’s (2020) experimental study on careless responding.

Our analyses show that RPR is under many conditions a significantly better careless response indicator than the conventional PR, no matter whether computer- or human-generated careless response patterns need to be detected. This result was in line with our expectation and confirms the as-yet untested proposition (e.g., Curran, 2016, pp. 9–10; Ward & Meade, 2023, p. 587) that calculating PR with different sets of scale halves and averaging the individual PRs to an overall measure (i.e., the RPR) results in a careless response indicator that is more accurate than the conventional PR measure, which is only based on a single set of scale half pairs—typically a set of even–odd scale half pairs.

When using RPR instead of PR, we expected the gain in detection accuracy to be larger under presumably “rough” conditions (i.e., only few facets with high item-specific error can be used when detecting invariant partial careless responding) than under presumably favorable conditions (i.e., many few facets with low item-specific error can be used when detecting uniform random full careless responding). The only support for this interaction hypothesis is indicated by the three-way interaction in the simulation study between the PR predictor, the number of facets, and the error per facet. This result suggests that using RPR instead of PR is associated with a relatively constant gain in detection accuracy that only decreases under conditions in which PR and RPR reach the maximal level of detection accuracy (i.e., an AUC value of 1).

### Recommendations

Across all conditions in the simulation study, the AUC increased from .789 to .833–.834 and the sensitivity from .494 to .558–.561 when using an RPR version instead of the conventional PR measure. Moreover, in all the simulation conditions that we examined, the averaged condition-specific detection accuracy of RPR versions never fell below the accuracy of the conventional PR measure. Thus, using an RPR version instead of PR only brings advantages. In the worst case, researchers obtain a PR measure that is less arbitrary in its computation and only as accurate as the conventional PR (i.e., even–odd consistency). In the best and more likely case, researchers obtain a PR measure that is less arbitrary in its computation and more accurate than the conventional PR measure. We therefore generally recommend using RPR instead of the conventional PR measure when screening questionnaire data for careless responding.

Our results also show that the three RPR versions examined, which were based on a different number of independently drawn resamples (i.e., 25, 50, 100), resulted in almost identical AUC and sensitivity values. Thus, using 25 resamples for the RPR computation was sufficient to obtain the expected gain in detection accuracy over the conventional PR measure, and using more resamples (i.e., 50 or 100) was not associated with any substantial further improvement in AUC and sensitivity values. We therefore recommend using 25 resamples (i.e., 25 different sets of scale half pairings) when calculating RPR. Furthermore, we recommend using the arithmetic mean for summarizing the individual PR values (i.e., calculating a mean-based RPR). As our supplementary analyses showed, this type of RPR calculation is generally more accurate than other types of RPR calculation that use different ways of summarizing the individual PR values (i.e., using the median or the standard deviation).

Finally, our results show how researchers can improve the detection accuracy of RPR. First, researchers should use validated questionnaires (assuming that such questionnaires include items that are more reliable than those of unvalidated questionnaires) and/or questionnaires with 15 or more facets. Second, our supplementary analyses showed that higher detection accuracy of RPR can be obtained when items are presented in a random rather than a fixed construct-wise order, especially when partial invariant careless responding needs to be detected.^6^ However, using longer and validated questionnaires and presenting their items in a random order may not be possible in every research context. Thus, whenever shorter or less validated questionnaires are used or a fixed construct-wise item presentation mode is chosen, we recommend the following procedure when using RPR for careless responding detection: First, instead on blindly relying on heuristics (e.g., screening RPR below .3; DeSimone et al., 2015; Zickar & Keith, 2023), researchers should apply (more conservative) cutoff values that match the measurement conditions of their study when screening the response protocols for careless responding according to the RPR values. For instance, to ensure a false-positive rate of 5% in our simulation, a cutoff value of .56 had to be used in the context of 15 facets with low item-specific error, but a cutoff value of −0.09 to −0.10 in the context of 15 facets with mediocre item-specific error (see Table 7). Second, researchers should use additional careless response indices that are less affected by the factors “number of facets” and “item-specific error per facet,” such as response time per item or Mahalanobis distance.

### Limitations and future research directions

Our study has several limitations. First, the conclusions drawn based on our simulation results are valid only for the conditions that we examined. We manipulated number of facets, extent of item-specific error per facet, type of careless responding, and severity of careless responding in the careless response protocols―factors that we thought would most likely affect the detection accuracy of PR and RPR. However, other factors that we did not examine may have a potential effect on the detection performance of PR and RPR, such as number of response options (e.g., 4, 5, 6), number of items per facet (e.g., 4, 6, 10), type of item keying (i.e., unidirectional vs. bidirectional), and type of item distribution (e.g., normal vs. skewed). Furthermore, we did not examine how challenges that are typically encountered in applied research settings, such as missing data, lack of unidimensionality of the scales and facets used, or different item functioning affect the performance of the PR and RPR.^7^^7^ Thus, future simulation studies should extend our work and examine whether factors that we held constant across our conditions have an effect on the detection accuracy of PR and RPR. In addition, it would be worthwhile to examine whether adaptations of RPR to the context of a single unidimensional scale also turn out to be useful in detecting careless responding.^8^

Second, we used latent factor models for the data generation because this model type allowed us to readily manipulate the factors that we examined in our study. However, our simulation results may not hold if other model types are used for data generation, such as models that are based on item response theory (IRT). Future simulation studies should examine whether our results can be replicated when IRT-based models, such as Rasch rating scale models (Andrich, 1978) or graded response models (Samejima, 1969), are used for data generation.

Third, we focused on comparing the detection performance of PR with that of the three RPR versions. With this focus, however, we left the question unanswered as to how RPR performs compared to other established careless response indices. Future studies could therefore look at how the RPR performs compared to average response time per item and Mahalanobis distance (e.g., Goldammer et al., 2020) and explore which of these indices best complements RPR.

### Conclusion

Screening questionnaire data for careless responding is important to ensure the credibility of study findings. However, screening will only be effective if researchers apply the most accurate indices and know under what conditions the applied indices perform favorably and less favorably. In our two studies, we examined the detection accuracy of RPR, which turns out to perform even better than the well-performing conventional PR. Further, our studies indicate that RPR performs the best when 15 or more facets with low item-specific error per facet are used, and the worst (but nevertheless acceptably) when only five facets with high item-specific error per facet can be used for calculation. These valuable insights may help applied researchers in more effective design of their careless response screening.

## Supplementary Information

Below is the link to the electronic supplementary material.

Supplementary file1 (DOCX 195 KB)

## Data Availability

The code to run the simulation and the data of the two studies are available under the following link: https://polybox.ethz.ch/index.php/s/OUeUYS9ZHkyu7YB

## Footnotes

1 The data-generating process (DGP) for this careful response protocol is identical to DGP used in some conditions of the simulation study (i.e., latent domain intercorrelation: .3; unstandardized item loadings: 1; unstandardized random normal error variance: .50).

2 A reviewer wondered why we had chosen the arithmetic mean for summarizing the different PRs and not another measure of central tendency. Because the literature offers little guidance in this regard, using the arithmetic mean was our best guess. However, we viewed the reviewer’s point as legitimate and therefore ran supplementary analyses in which we examined whether using a median-based RPR would alter the results. In these analyses, however, the mean-based RPR turned out to be a slightly better index for careless responding than the median-based RPR (see supplementary material, Tables S1–S3). Across all conditions in the simulation study, the AUC increased from .824–.827 to .833–.834 and the sensitivity from .552–.557 to .558–.561 when using a mean-based RPR version instead of a median-based RPR version. Moreover, in all the simulation conditions that we examined, the averaged condition-specific detection accuracy of mean-based RPR versions never fell below the accuracy of their median-based RPR counterparts. Thus, we recommend using a mean-based rather than a median-based RPR version for detecting careless responding.

3 Based on the numerical example in Table 3, a reviewer wondered whether the PR values of careful respondents would generally be in a tighter range than those of careless respondents and thus could be an informative careless response index as well—potentially, an even more informative index than the (arithmetic) mean-based RPR. To examine this question, we ran supplementary analyses in which we examined whether using the standard deviation of PR values would alter the results. In these supplementary analyses, the standard deviation of PR values turned out to perform satisfactorily in most conditions (i.e., better than chance), which confirmed the reviewer’s hypothesis that the PR values of careful respondents are in a tighter range than those of careless respondents. However, the detection accuracies of the standard deviation of the PR values almost never reached those of the mean-based RPRs (see supplementary material, Tables S4–S6). Across all conditions in the simulation study, the AUC increased from .802–.811 to .833–.834 and the sensitivity from .518–.534 to .558–.561 when using a mean-based RPR version instead of a version of that is based on the standard deviation of the PR values. Moreover, in all but two simulation conditions that we examined, the averaged condition-specific detection accuracy of mean-based RPR versions never fell below the accuracy of their “standard deviation counterparts.” Thus, the mean-based RPR may be considered as more informative than the standard deviation of PR values and should therefore be preferably used for careless responding detection.

4 A reviewer wondered how the random selection and replacement of items with careless responses is in line with the typical phenomenon of back careless responding, where the careless responses are concentrated at the end of the questionnaire (Yu & Cheng, 2019). We were aware of the research that shows that partial careless responding most likely occurs at the end of the survey, and we also aimed to represent this case with our partial careless responding condition. However, we had a specific questionnaire setup in mind when programming the partial careless responding condition in our simulation—the case in which the order of item display is random. This item presentation mode seems to be a legitimate choice for non-cognitive items (Bandalos, 2018, p. 222) and may also come with the advantage of a better fitting confirmatory measurement model, due to reduced residual correlations among the items (Bandalos, 2021), Thus, in our partial careless responding conditions, those items that were randomly selected and replaced by careless response patterns can be thought of as being placed at the end of the survey. Furthermore, the case of random item order further means that each item of each construct has the equal probability of being placed in the last half of the questionnaire and thus an equal probability of being affected by back careless responding. Nevertheless, there may be cases where researchers may wish to display the items in a fixed construct-wise order. In such a case, only constructs and their items that are placed in the last half of the questionnaire will be affected by careless responding. Eventually, we realized that such a fixed construct-wise item presentation mode may also have an effect on the performance of the PR and RPR. We therefore ran a supplementary simulation study with the identical settings as outlined in the paper but changed the item selection procedure for the partial careless responding conditions (i.e., the last 50% of items were replaced in each careless response protocol). For most conditions, the detection accuracies obtained from the supplementary simulation study were comparable to those that we obtained from the simulation study reported in the paper. However, in conditions where partial invariant careless responding had to be detected, the performance of the PR and RPRs differed substantially across the two simulations. Whereas the PR and RPRs had satisfactory to good detection accuracies for detecting partial invariant careless responding when items that were randomly selected were replaced by careless responses (i.e., representing back careless responding in a questionnaire in which items are randomly displayed), they performed completely unsatisfactorily (i.e., they were more indicative of careful responding) when the last 50% of items were replaced with careless responses (i.e., representing back careless responding in a questionnaire with a fixed construct-wise item order) (see supplementary material, Tables S7–S15). Thus, whenever possible, researchers should choose a random item presentation mode over a fixed construct-wise item presentation mode, because higher detection accuracies can be achieved when using PR and RPRs.

5 As a robustness check, we reran the analyses for the comparisons between PR and RPR with cluster-robust standard error estimation (using replication id as cluster variable in linear regression models). In these additional analyses, the test statistics of nearly all pairwise comparisons among the RPR versions reached statistical significance, even though their AUC and sensitivity values only differed at the third decimal place. However, the effect sizes that were associated with each of the pairwise comparisons confirmed our conclusion (Cohen’s *d* for AUC_RPRs vs PR_ = 0.33–0.34, Cohen’s *d* for AUC_RPRs vs RPRs_ = 0.007–0.010, Cohen’s *d* for Sensitivity_RPRs vs PR_ = 0.21–0.23, Cohen’s *d* for Sensitivity_RPRs vs RPRs_ = 0.009–0.011) that the differences between the RPR versions and the conventional PR (e.g., RPR25 vs. PR) may be considered as practically meaningful and those between the different RPR versions (e.g. RPR100 vs. RPR25) as neglectable.

6 According to Goldammer et al., 2024, another approach for improving the detection accuracy of RPR would be to use scales that contain bidirectionally keyed items (i.e., positively and negatively worded items) instead of scales that are only composed of unidirectionally keyed items (i.e., only positively or only negatively worded items).

7 We expect PR and RPR to be more accurate in detecting careless responding if scales are used for which unidimensionality and lack of different item functioning (across subgroups) can be assumed. However, if researchers doubt the quality of their scales, they should explicitly test for these two preconditions.

8 One of the three following strategies might be used to adapt PR and RPR to the context of a single unidimensional scale. First, researchers could split the items of the scale into three or more pairs of item blocks and calculate the within-person correlation across these pairs of items blocks. This version may be repeated across a series randomly rearranged set of pairs of item blocks and is most in line with the original spirit of the resampled personal reliability. However, a sufficient number of items needs to be given (e.g., 12 items or more) to obtain block scores that are really different from the item scores. Second, researchers could calculate the within-person correlation across each item pair combination, which would result in a statistic that is very similar to the semantic synonym index (Curran, 2016, p. 9). Third, researchers could calculate the within-person correlation across each item–rest combination. As this approach examines whether item responses are in line with the score of the remaining items, it may have certain similarities with the Guttman error index (see Niessen et al., 2016, pp. 4, 10).

## Declarations

### Transparency

The studies’ designs, hypotheses, and analyses were not preregistered.
