# Supplementary material for: A comparison of conventional and resampled personal reliability in detecting careless responding
Source: Behav Res Methods. 2024 Sep 16;56(8):8831–51. doi: 10.3758/s13428-024-02506-0 (PMC11525424; doi:10.3758/s13428-024-02506-0)
Supplement: Supplementary file 1 — Supplementary file1 (DOCX 195 KB) [file 13428_2024_2506_MOESM1_ESM.docx]

# Supplementary Material for:

# A Comparison of Conventional and Resampled Personal Reliability in Detecting Careless Responding

## Randomly Selected Items Replaced by Careless Response Patterns in the Partial Careless Responding Condition

Like in the main paper, the results of the following two sections are based on randomly selected items for the partial careless responding conditions. This meant that we randomly selected and replaced 50% of the items of every partial careless response protocol with either invariant or uniform careless responses. This represents the case where the order of item display is random. and those items that were randomly selected and replaced by careless response patterns can be thought of as being placed at the end of the survey.

### Median-Based Resampled Personal Reliability Measures

Tables S1 to S3 show the results when the median is used for computing the resampled personal reliability (RPR).

#### Table S1

*Area Under the Curve for the Conventional Personal Reliability and Three Resampled Personal Reliability Versions Across Simulation*

| Error per facet | Type of CR | PR | RPR25 | RPR50 | RPR100 |
| --- | --- | --- | --- | --- | --- |
| Full careless responding | | | | | |
| 5 facets in survey |  |  |  |  |  |
| low | invariant | 0.839 (0.024) | 0.884 (0.021) | 0.890 (0.020) | 0.891 (0.020) |
| low | uniform | 0.844 (0.024) | 0.898 (0.017) | 0.901 (0.017) | 0.902 (0.017) |
| mediocre | invariant | 0.655 (0.031) | 0.699 (0.031) | 0.702 (0.031) | 0.702 (0.032) |
| mediocre | uniform | 0.662 (0.036) | 0.711 (0.034) | 0.714 (0.034) | 0.715 (0.034) |
| high | invariant | 0.582 (0.034) | 0.615 (0.035) | 0.619 (0.035) | 0.619 (0.035) |
| high | uniform | 0.584 (0.033) | 0.623 (0.033) | 0.625 (0.032) | 0.626 (0.033) |
| 15 facets in survey |  |  |  |  |  |
| low | invariant | 0.979 (0.008) | 0.994 (0.003) | 0.994 (0.003) | 0.994 (0.003) |
| low | uniform | 0.979 (0.008) | 0.994 (0.003) | 0.994 (0.003) | 0.994 (0.003) |
| mediocre | invariant | 0.830 (0.026) | 0.878 (0.019) | 0.880 (0.019) | 0.881 (0.019) |
| mediocre | uniform | 0.828 (0.024) | 0.876 (0.021) | 0.879 (0.021) | 0.879 (0.021) |
| high | invariant | 0.712 (0.033) | 0.753 (0.033) | 0.755 (0.033) | 0.756 (0.033) |
| high | uniform | 0.716 (0.030) | 0.759 (0.025) | 0.761 (0.025) | 0.762 (0.025) |
| 30 facets in survey |  |  |  |  |  |
| low | invariant | 0.999 (0.001) | 1 (0) | 1 (0) | 1 (0) |
| low | uniform | 0.999 (0.001) | 1 (0) | 1 (0) | 1 (0) |
| mediocre | invariant | 0.916 (0.019) | 0.954 (0.012) | 0.955 (0.012) | 0.956 (0.012) |
| mediocre | uniform | 0.917 (0.015) | 0.953 (0.011) | 0.954 (0.011) | 0.955 (0.011) |
| high | invariant | 0.799 (0.028) | 0.844 (0.026) | 0.846 (0.025) | 0.846 (0.026) |
| high | uniform | 0.800 (0.030) | 0.846 (0.025) | 0.849 (0.025) | 0.849 (0.025) |
| Partial careless responding | | | | | |
| 5 facets in survey |  |  |  |  |  |
| low | invariant | 0.765 (0.032) | 0.825 (0.024) | 0.828 (0.024) | 0.828 (0.024) |
| low | uniform | 0.819 (0.024) | 0.872 (0.021) | 0.874 (0.020) | 0.876 (0.021) |
| mediocre | invariant | 0.610 (0.039) | 0.643 (0.037) | 0.645 (0.035) | 0.645 (0.036) |
| mediocre | uniform | 0.632 (0.031) | 0.676 (0.032) | 0.678 (0.033) | 0.678 (0.032) |
| high | invariant | 0.550 (0.037) | 0.566 (0.037) | 0.570 (0.037) | 0.569 (0.037) |
| high | uniform | 0.564 (0.038) | 0.593 (0.030) | 0.597 (0.033) | 0.597 (0.034) |
| 15 facets in survey |  |  |  |  |  |
| low | invariant | 0.930 (0.016) | 0.964 (0.010) | 0.965 (0.010) | 0.966 (0.010) |
| low | uniform | 0.967 (0.009) | 0.987 (0.006) | 0.987 (0.006) | 0.987 (0.006) |
| mediocre | invariant | 0.731 (0.031) | 0.777 (0.028) | 0.779 (0.028) | 0.780 (0.028) |
| mediocre | uniform | 0.774 (0.029) | 0.822 (0.026) | 0.825 (0.026) | 0.825 (0.026) |
| high | invariant | 0.647 (0.038) | 0.674 (0.030) | 0.676 (0.030) | 0.676 (0.03) |
| high | uniform | 0.671 (0.031) | 0.708 (0.031) | 0.710 (0.032) | 0.711 (0.031) |
| 30 facets in survey |  |  |  |  |  |
| low | invariant | 0.983 (0.007) | 0.994 (0.003) | 0.995 (0.003) | 0.995 (0.003) |
| low | uniform | 0.996 (0.002) | 0.999 (0.001) | 0.999 (0.001) | 0.9995 (0.001) |
| mediocre | invariant | 0.813 (0.03) | 0.862 (0.026) | 0.864 (0.024) | 0.864 (0.025) |
| mediocre | uniform | 0.872 (0.020) | 0.917 (0.015) | 0.918 (0.015) | 0.919 (0.015) |
| high | invariant | 0.693 (0.033) | 0.731 (0.030) | 0.732 (0.029) | 0.734 (0.029) |
| high | uniform | 0.742 (0.028) | 0.785 (0.025) | 0.787 (0.026) | 0.788 (0.026) |

*Note.* Numbers presented are means of areas under the receiver operating characteristic curves across the 100 replications per condition (*SD*s in parentheses). Type of CR = type of careless responding; PR = conventional personal reliability (i.e., even-odd consistency); RPR25 = median-based resampled personal reliability with 25 sets of scale half pairings; RPR50 = median-based resampled personal reliability with 50 sets of scale half pairings; RPR100 = median-based resampled personal reliability with 100 sets of scale half pairings; low = low item error per facet with normally distributed errors with *N*(0, 0.5) for all items of each factor; mediocre = mediocre item error per facet with normally distributed errors with *N*(0, 0.5) for one half of the items of each factor and normally distributed errors with *N*(0, 1.5) for the other half of the items of each factor; high = high item error per facet with normally distributed errors with *N*(0, 1.5) for all items of each factor; invariant = invariant careless responding; uniform = uniform random careless responding; Full careless responding = all item responses of the response protocol were replaced with simulated careless responses; Partial careless responding = 50% of the item responses of the response protocol were randomly selected and replaced with simulated careless responses.

#### Table S2

*Sensitivity at a False Positive Rate of 5% for the Conventional Personal Reliability and Three Resampled Personal Reliability Versions Across Simulation Conditions*

| Error per facet | Type of CR | PR | RPR25 | RPR50 | RPR100 |
| --- | --- | --- | --- | --- | --- |
| Full careless responding | | | | | |
| 5 facets in survey |  |  |  |  |  |
| low | invariant | 0.413 (0.074) | 0.465 (0.095) | 0.477 (0.089) | 0.480 (0.091) |
| low | uniform | 0.435 (0.087) | 0.497 (0.083) | 0.497 (0.081) | 0.500 (0.077) |
| mediocre | invariant | ^a^ | ^a^ | ^a^ | ^a^ |
| mediocre | uniform | ^a^ | 0.333 (0) _[1]_ | 0.261 (0.039) _[2]_ | 0.311 (0.019) _[3]_ |
| high | invariant | ^a^ | ^a^ | ^a^ | ^a^ |
| high | uniform | ^a^ | ^a^ | ^a^ | ^a^ |
| 15 facets in survey |  |  |  |  |  |
| low | invariant | 0.896 (0.046) | 0.973 (0.022) | 0.976 (0.020) | 0.977 (0.019) |
| low | uniform | 0.901 (0.042) | 0.977 (0.017) | 0.975 (0.018) | 0.978 (0.015) |
| mediocre | invariant | 0.386 (0.069) | 0.462 (0.079) | 0.460 (0.083) | 0.464 (0.082) |
| mediocre | uniform | 0.384 (0.070) | 0.459 (0.090) | 0.463 (0.091) | 0.472 (0.087) |
| high | invariant | 0.201 (0.066) | 0.220 (0.072) | 0.225 (0.078) | 0.224 (0.079) |
| high | uniform | 0.204 (0.060) | 0.235 (0.058) | 0.245 (0.067) | 0.244 (0.065) |
| 30 facets in survey |  |  |  |  |  |
| low | invariant | 0.996 (0.007) | 0.9996 (0.003) | 0.9996 (0.003) | 0.9997 (0.002) |
| low | uniform | 0.994 (0.007) | 0.9999 (0.001) | 0.9999 (0.001) | 0.9999 (0.001) |
| mediocre | invariant | 0.620 (0.077) | 0.751 (0.074) | 0.755 (0.073) | 0.759 (0.072) |
| mediocre | uniform | 0.629 (0.069) | 0.750 (0.069) | 0.755 (0.068) | 0.763 (0.066) |
| high | invariant | 0.321 (0.071) | 0.385 (0.078) | 0.394 (0.082) | 0.391 (0.085) |
| high | uniform | 0.322 (0.081) | 0.391 (0.094) | 0.404 (0.089) | 0.407 (0.091) |
| Partial careless responding | | | | | |
| 5 facets in survey |  |  |  |  |  |
| low | invariant | 0.287 (0.063) | 0.310 (0.072) | 0.311 (0.068) | 0.314 (0.064) |
| low | uniform | 0.368 (0.071) | 0.404 (0.083) | 0.404 (0.084) | 0.405 (0.081) |
| mediocre | invariant | ^a^ | 0.233 (0.011) _[3]_ | 0.196 (0.006) _[3]_ | 0.200 (0.016) _[2]_ |
| mediocre | uniform | ^a^ | ^a^ | 0.256 (0) _[2]_ | 0.283 (0.008) _[2]_ |
| high | invariant | ^a^ | ^a^ | ^a^ | ^a^ |
| high | uniform | ^a^ | ^a^ | ^a^ | ^a^ |
| 15 facets in survey |  |  |  |  |  |
| low | invariant | 0.691 (0.073) | 0.805 (0.067) | 0.816 (0.063) | 0.821 (0.061) |
| low | uniform | 0.834 (0.055) | 0.933 (0.039) | 0.935 (0.037) | 0.937 (0.040) |
| mediocre | invariant | 0.228 (0.064) | 0.263 (0.069) | 0.265 (0.066) | 0.268 (0.069) |
| mediocre | uniform | 0.278 (0.066) | 0.337 (0.082) | 0.344 (0.077) | 0.340 (0.074) |
| high | invariant | 0.132 (0.048) | 0.142 (0.048) | 0.143 (0.048) | 0.143 (0.049) |
| high | uniform | 0.159 (0.054) | 0.172 (0.063) | 0.177 (0.061) | 0.176 (0.065) |
| 30 facets in survey |  |  |  |  |  |
| low | invariant | 0.917 (0.034) | 0.972 (0.02) | 0.974 (0.018) | 0.976 (0.018) |
| low | uniform | 0.985 (0.014) | 0.999 (0.003) | 0.999 (0.003) | 0.999 (0.003) |
| mediocre | invariant | 0.353 (0.072) | 0.437 (0.084) | 0.444 (0.083) | 0.449 (0.087) |
| mediocre | uniform | 0.481 (0.079) | 0.604 (0.078) | 0.605 (0.082) | 0.605 (0.079) |
| high | invariant | 0.190 (0.058) | 0.213 (0.063) | 0.212 (0.065) | 0.215 (0.061) |
| high | uniform | 0.237 (0.060) | 0.291 (0.070) | 0.291 (0.071) | 0.299 (0.067) |

*Note.* Numbers presented are means of areas under the receiver operating characteristic curves across the 100 replications per condition (*SD*s in parentheses). For some conditions, however, we could not achieve 100 successful replications. For these conditions, the number of successful replications is shown in squared brackets and in lowercase. Type of CR = type of careless responding; PR = conventional personal reliability (i.e., even-odd consistency); RPR25 = median-based resampled personal reliability with 25 sets of scale half pairings; RPR50 = median-based resampled personal reliability with 50 sets of scale half pairings; RPR100 = median-based resampled personal reliability with 100 sets of scale half pairings; low = low item error per facet with normally distributed errors with *N*(0, 0.5) for all items of each factor; mediocre = mediocre item error per facet with normally distributed errors with *N*(0, 0.5) for one half of the items of each factor and normally distributed errors with *N*(0, 1.5) for the other half of the items of each factor; high = high item error per facet with normally distributed errors with *N*(0, 1.5) for all items of each factor; invariant = invariant careless responding; uniform = uniform random careless responding; Full careless responding = all item responses of the response protocol were replaced with simulated careless responses; Partial careless responding = 50% of the item responses of the response protocol were randomly selected and replaced with simulated careless responses.

^a^ Sensitivities could not be computed because of missing values in the matrix (i.e., non-zero sensitivities could only be calculated for false positive rates that were higher than the 5% level that we aimed for).

#### Table S3

*Cut-Off Values at a False Positive Rate of 5% for the Conventional Personal Reliability and Three Resampled Personal Reliability Versions Across Simulation Conditions*

| Error per facet | Type of CR | PR | RPR25 | RPR50 | RPR100 |
| --- | --- | --- | --- | --- | --- |
| Full careless responding | | | | | |
| 5 facets in survey |  |  |  |  |  |
| low | invariant | -0.257 (0.249) | -0.100 (0.211) | -0.070 (0.207) | -0.066 (0.207) |
| low | uniform | -0.310 (0.246) | -0.103 (0.22) | -0.110 (0.214) | -0.088 (0.208) |
| mediocre | invariant | -1 (0) | -1 (0) | -1 (0) | -1 (0) |
| mediocre | uniform | -1 (0) | -0.9997 (0.003) | -0.9996 (0.003) | -0.998 (0.011) |
| high | invariant | -1 (0) | -1 (0) | -1 (0) | -1 (0) |
| high | uniform | -1 (0) | -1 (0) | -1 (0) | -1 (0) |
| 15 facets in survey |  |  |  |  |  |
| low | invariant | 0.515 (0.047) | 0.572 (0.034) | 0.575 (0.033) | 0.574 (0.032) |
| low | uniform | 0.518 (0.043) | 0.572 (0.033) | 0.573 (0.035) | 0.574 (0.033) |
| mediocre | invariant | -0.200 (0.095) | -0.073 (0.076) | -0.069 (0.072) | -0.068 (0.074) |
| mediocre | uniform | -0.209 (0.102) | -0.082 (0.079) | -0.077 (0.080) | -0.073 (0.080) |
| high | invariant | -0.647 (0.140) | -0.478 (0.116) | -0.474 (0.119) | -0.467 (0.117) |
| high | uniform | -0.640 (0.144) | -0.461 (0.112) | -0.448 (0.120) | -0.438 (0.119) |
| 30 facets in survey |  |  |  |  |  |
| low | invariant | 0.638 (0.020) | 0.668 (0.017) | 0.670 (0.017) | 0.670 (0.017) |
| low | uniform | 0.639 (0.023) | 0.666 (0.018) | 0.668 (0.018) | 0.669 (0.017) |
| mediocre | invariant | 0.095 (0.048) | 0.177 (0.042) | 0.179 (0.041) | 0.180 (0.040) |
| mediocre | uniform | 0.106 (0.051) | 0.179 (0.040) | 0.179 (0.040) | 0.184 (0.039) |
| high | invariant | -0.203 (0.073) | -0.109 (0.058) | -0.101 (0.052) | -0.103 (0.053) |
| high | uniform | -0.208 (0.077) | -0.114 (0.063) | -0.100 (0.057) | -0.101 (0.058) |
| Partial careless responding | | | | | |
| 5 facets in survey |  |  |  |  |  |
| low | invariant | -0.324 (0.291) | -0.103 (0.232) | -0.088 (0.209) | -0.078 (0.218) |
| low | uniform | -0.323 (0.274) | -0.146 (0.243) | -0.143 (0.229) | -0.141 (0.241) |
| mediocre | invariant | -1 (0) | -0.997 (0.018) | -0.997 (0.025) | -0.999 (0.007) |
| mediocre | uniform | -1 (0) | -1 (0) | -0.999 (0.005) | -0.999 (0.008) |
| high | invariant | -1 (0) | -1 (0) | -1 (0) | -1 (0) |
| high | uniform | -1 (0) | -1 (0) | -1 (0) | -1 (0) |
| 15 facets in survey |  |  |  |  |  |
| low | invariant | 0.522 (0.045) | 0.571 (0.037) | 0.575 (0.036) | 0.575 (0.037) |
| low | uniform | 0.514 (0.039) | 0.571 (0.036) | 0.573 (0.037) | 0.575 (0.035) |
| mediocre | invariant | -0.215 (0.101) | -0.076 (0.086) | -0.069 (0.081) | -0.067 (0.083) |
| mediocre | uniform | -0.227 (0.101) | -0.090 (0.084) | -0.078 (0.074) | -0.082 (0.073) |
| high | invariant | -0.627 (0.127) | -0.455 (0.117) | -0.452 (0.106) | -0.450 (0.114) |
| high | uniform | -0.656 (0.158) | -0.488 (0.129) | -0.475 (0.117) | -0.469 (0.123) |
| 30 facets in survey |  |  |  |  |  |
| low | invariant | 0.637 (0.021) | 0.666 (0.016) | 0.668 (0.017) | 0.669 (0.018) |
| low | uniform | 0.639 (0.018) | 0.671 (0.016) | 0.670 (0.014) | 0.671 (0.015) |
| mediocre | invariant | 0.095 (0.051) | 0.171 (0.045) | 0.177 (0.045) | 0.179 (0.045) |
| mediocre | uniform | 0.105 (0.054) | 0.179 (0.048) | 0.181 (0.046) | 0.179 (0.047) |
| high | invariant | -0.208 (0.081) | -0.105 (0.060) | -0.105 (0.062) | -0.100 (0.059) |
| high | uniform | -0.195 (0.065) | -0.091 (0.055) | -0.090 (0.053) | -0.085 (0.051) |

*Note.* Numbers presented are means of the cut-off values at a false-positive rate of 5 % across the 100 replications per condition (*SD*s in parentheses). Type of CR = type of careless responding; PR = conventional personal reliability (i.e., even-odd consistency); RPR25 = median-based resampled personal reliability with 25 sets of scale half pairings; RPR50 = median-based resampled personal reliability with 50 sets of scale half pairings; RPR100 = median-based resampled personal reliability with 100 sets of scale half pairings; low = low item error per facet with normally distributed errors with *N*(0, 0.5) for all items of each factor; mediocre = mediocre item error per facet with normally distributed errors with *N*(0, 0.5) for one half of the items of each factor and normally distributed errors with *N*(0, 1.5) for the other half of the items of each factor; high = high item error per facet with normally distributed errors with *N*(0, 1.5) for all items of each factor; invariant = invariant careless responding; uniform = uniform random careless responding; Full careless responding = all item responses of the response protocol were replaced with simulated careless responses; Partial careless responding = 50% of the item responses of the response protocol were randomly selected and replaced with simulated careless responses.

### Standard Deviation of the Personal Reliabilities as Detection Measure

Tables S4 to S6 show the results when the standard deviation of the personal reliability (PR) values is used as detection measure.

#### Table S4

*Area Under the Curve for the Conventional Personal Reliability and Three Resampled Personal Reliability Versions Across Simulation Conditions*

| Error per facet | Type of CR | PR | STDEV_RPR25 | STDEV_RPR50 | STDEV_RPR100 |
| --- | --- | --- | --- | --- | --- |
| Full careless responding | | | | | |
| 5 facets in survey |  |  |  |  |  |
| low | invariant | 0.839 (0.024) | 0.777 (0.030) | 0.787 (0.028) | 0.790 (0.029) |
| low | uniform | 0.844 (0.024) | 0.849 (0.025) | 0.861 (0.024) | 0.865 (0.025) |
| mediocre | invariant | 0.655 (0.031) | 0.614 (0.035) | 0.619 (0.035) | 0.622 (0.036) |
| mediocre | uniform | 0.662 (0.036) | 0.682 (0.038) | 0.691 (0.034) | 0.695 (0.035) |
| high | invariant | 0.582 (0.034) | 0.518 (0.037) | 0.524 (0.036) | 0.524 (0.035) |
| high | uniform | 0.584 (0.033) | 0.585 (0.030) | 0.588 (0.030) | 0.590 (0.029) |
| 15 facets in survey |  |  |  |  |  |
| low | invariant | 0.979 (0.008) | 0.980 (0.010) | 0.985 (0.008) | 0.988 (0.006) |
| low | uniform | 0.979 (0.008) | 0.981 (0.009) | 0.985 (0.008) | 0.987 (0.007) |
| mediocre | invariant | 0.830 (0.026) | 0.872 (0.022) | 0.880 (0.020) | 0.884 (0.020) |
| mediocre | uniform | 0.828 (0.024) | 0.867 (0.022) | 0.875 (0.022) | 0.878 (0.021) |
| high | invariant | 0.712 (0.033) | 0.711 (0.035) | 0.716 (0.036) | 0.721 (0.033) |
| high | uniform | 0.716 (0.030) | 0.709 (0.030) | 0.717 (0.027) | 0.722 (0.029) |
| 30 facets in survey |  |  |  |  |  |
| low | invariant | 0.999 (0.001) | 0.998 (0.004) | 0.999 (0.003) | 0.999 (0.002) |
| low | uniform | 0.999 (0.001) | 0.998 (0.003) | 0.999 (0.003) | 0.999 (0.003) |
| mediocre | invariant | 0.916 (0.019) | 0.951 (0.013) | 0.958 (0.013) | 0.962 (0.012) |
| mediocre | uniform | 0.917 (0.015) | 0.948 (0.012) | 0.957 (0.010) | 0.960 (0.010) |
| high | invariant | 0.799 (0.028) | 0.801 (0.029) | 0.815 (0.026) | 0.820 (0.026) |
| high | uniform | 0.800 (0.030) | 0.807 (0.027) | 0.819 (0.027) | 0.824 (0.028) |
| Partial careless responding | | | | | |
| 5 facets in survey |  |  |  |  |  |
| low | invariant | 0.765 (0.032) | 0.810 (0.027) | 0.818 (0.026) | 0.820 (0.026) |
| low | uniform | 0.819 (0.024) | 0.833 (0.023) | 0.842 (0.021) | 0.846 (0.020) |
| mediocre | invariant | 0.610 (0.039) | 0.650 (0.037) | 0.657 (0.035) | 0.659 (0.035) |
| mediocre | uniform | 0.632 (0.031) | 0.662 (0.034) | 0.668 (0.033) | 0.672 (0.034) |
| high | invariant | 0.550 (0.037) | 0.559 (0.036) | 0.561 (0.037) | 0.562 (0.036) |
| high | uniform | 0.564 (0.038) | 0.575 (0.036) | 0.579 (0.035) | 0.579 (0.035) |
| 15 facets in survey |  |  |  |  |  |
| low | invariant | 0.930 (0.016) | 0.960 (0.011) | 0.963 (0.010) | 0.965 (0.010) |
| low | uniform | 0.967 (0.009) | 0.975 (0.011) | 0.977 (0.010) | 0.979 (0.009) |
| mediocre | invariant | 0.731 (0.031) | 0.799 (0.027) | 0.803 (0.028) | 0.807 (0.027) |
| mediocre | uniform | 0.774 (0.029) | 0.827 (0.024) | 0.833 (0.024) | 0.837 (0.024) |
| high | invariant | 0.647 (0.038) | 0.658 (0.029) | 0.664 (0.029) | 0.664 (0.030) |
| high | uniform | 0.671 (0.031) | 0.673 (0.033) | 0.681 (0.031) | 0.682 (0.031) |
| 30 facets in survey |  |  |  |  |  |
| low | invariant | 0.983 (0.007) | 0.991 (0.005) | 0.993 (0.004) | 0.994 (0.003) |
| low | uniform | 0.996 (0.002) | 0.998 (0.002) | 0.998 (0.002) | 0.999 (0.002) |
| mediocre | invariant | 0.813 (0.030) | 0.878 (0.023) | 0.887 (0.023) | 0.893 (0.021) |
| mediocre | uniform | 0.872 (0.020) | 0.920 (0.015) | 0.929 (0.012) | 0.933 (0.012) |
| high | invariant | 0.693 (0.033) | 0.710 (0.030) | 0.720 (0.030) | 0.725 (0.029) |
| high | uniform | 0.742 (0.028) | 0.753 (0.028) | 0.761 (0.027) | 0.767 (0.027) |

*Note.* Numbers presented are means of areas under the receiver operating characteristic curves across the 100 replications per condition (*SD*s in parentheses). Type of CR = type of careless responding; PR = conventional personal reliability (i.e., even-odd consistency); STDEV_RPR25 = standard deviation among the personal reliability values that were obtained when calculating the resampled personal reliability with 25 sets of scale half pairings; STDEV_RPR50 = standard deviation among the personal reliability values that were obtained when calculating the resampled personal reliability with 50 sets of scale half pairings; STDEV_RPR100 = standard deviation among the personal reliability values that were obtained when calculating the resampled personal reliability with 100 sets of scale half pairings; low = low item error per facet with normally distributed errors with *N*(0, 0.5) for all items of each factor; mediocre = mediocre item error per facet with normally distributed errors with *N*(0, 0.5) for one half of the items of each factor and normally distributed errors with *N*(0, 1.5) for the other half of the items of each factor; high = high item error per facet with normally distributed errors with *N*(0, 1.5) for all items of each factor; invariant = invariant careless responding; uniform = uniform random careless responding; Full careless responding = all item responses of the response protocol were replaced with simulated careless responses; Partial careless responding = 50% of the item responses of the response protocol were randomly selected and replaced with simulated careless responses.

#### Table S5

*Sensitivity at a False Positive Rate of 5% for the Conventional Personal Reliability and Three Resampled Personal Reliability Versions Across Simulation Conditions*

| Error per facet | Type of CR | PR | STDEV_RPR25 | STDEV_RPR50 | STDEV_RPR100 |
| --- | --- | --- | --- | --- | --- |
| Full careless responding | | | | | |
| 5 facets in survey |  |  |  |  |  |
| low | invariant | 0.413 (0.074) | 0.310 (0.09) | 0.314 (0.092) | 0.305 (0.097) |
| low | uniform | 0.435 (0.087) | 0.311 (0.085) | 0.327 (0.095) | 0.323 (0.093) |
| mediocre | invariant | ^a^ | 0.140 (0.056) | 0.146 (0.052) | 0.156 (0.056) |
| mediocre | uniform | ^a^ | 0.133 (0.053) | 0.137 (0.057) | 0.145 (0.049) |
| high | invariant | ^a^ | 0.066 (0.033) | 0.070 (0.039) | 0.076 (0.037) |
| high | uniform | ^a^ | 0.071 (0.030) | 0.068 (0.030) | 0.072 (0.029) |
| 15 facets in survey |  |  |  |  |  |
| low | invariant | 0.896 (0.046) | 0.937 (0.029) | 0.947 (0.028) | 0.954 (0.026) |
| low | uniform | 0.901 (0.042) | 0.937 (0.030) | 0.950 (0.024) | 0.952 (0.025) |
| mediocre | invariant | 0.386 (0.069) | 0.459 (0.090) | 0.473 (0.084) | 0.489 (0.074) |
| mediocre | uniform | 0.384 (0.070) | 0.462 (0.091) | 0.471 (0.083) | 0.480 (0.083) |
| high | invariant | 0.201 (0.066) | 0.140 (0.056) | 0.138 (0.060) | 0.141 (0.058) |
| high | uniform | 0.204 (0.060) | 0.145 (0.052) | 0.144 (0.050) | 0.153 (0.056) |
| 30 facets in survey |  |  |  |  |  |
| low | invariant | 0.996 (0.007) | 0.997 (0.006) | 0.998 (0.005) | 0.999 (0.004) |
| low | uniform | 0.994 (0.007) | 0.995 (0.007) | 0.998 (0.005) | 0.998 (0.005) |
| mediocre | invariant | 0.620 (0.077) | 0.765 (0.064) | 0.796 (0.065) | 0.812 (0.064) |
| mediocre | uniform | 0.629 (0.069) | 0.752 (0.065) | 0.785 (0.057) | 0.802 (0.057) |
| high | invariant | 0.321 (0.071) | 0.302 (0.083) | 0.316 (0.081) | 0.330 (0.080) |
| high | uniform | 0.322 (0.081) | 0.317 (0.082) | 0.332 (0.078) | 0.341 (0.089) |
| Partial careless responding | | | | | |
| 5 facets in survey |  |  |  |  |  |
| low | invariant | 0.287 (0.063) | 0.289 (0.068) | 0.283 (0.080) | 0.284 (0.070) |
| low | uniform | 0.368 (0.071) | 0.272 (0.089) | 0.278 (0.089) | 0.267 (0.089) |
| mediocre | invariant | ^a^ | 0.130 (0.051) | 0.134 (0.042) | 0.142 (0.053) |
| mediocre | uniform | ^a^ | 0.122 (0.044) | 0.130 (0.046) | 0.131 (0.048) |
| high | invariant | ^a^ | 0.074 (0.032) | 0.075 (0.035) | 0.078 (0.029) |
| high | uniform | ^a^ | 0.068 (0.034) | 0.071 (0.031) | 0.073 (0.038) |
| 15 facets in survey |  |  |  |  |  |
| low | invariant | 0.691 (0.073) | 0.797 (0.058) | 0.808 (0.061) | 0.813 (0.058) |
| low | uniform | 0.834 (0.055) | 0.886 (0.049) | 0.895 (0.043) | 0.900 (0.049) |
| mediocre | invariant | 0.228 (0.064) | 0.311 (0.076) | 0.314 (0.071) | 0.332 (0.072) |
| mediocre | uniform | 0.278 (0.066) | 0.358 (0.078) | 0.367 (0.074) | 0.369 (0.076) |
| high | invariant | 0.132 (0.048) | 0.123 (0.042) | 0.124 (0.042) | 0.129 (0.046) |
| high | uniform | 0.159 (0.054) | 0.123 (0.048) | 0.123 (0.048) | 0.129 (0.051) |
| 30 facets in survey |  |  |  |  |  |
| low | invariant | 0.917 (0.034) | 0.955 (0.026) | 0.967 (0.022) | 0.969 (0.022) |
| low | uniform | 0.985 (0.014) | 0.992 (0.008) | 0.996 (0.007) | 0.996 (0.007) |
| mediocre | invariant | 0.353 (0.072) | 0.502 (0.084) | 0.530 (0.084) | 0.544 (0.081) |
| mediocre | uniform | 0.481 (0.079) | 0.636 (0.081) | 0.652 (0.073) | 0.673 (0.073) |
| high | invariant | 0.190 (0.058) | 0.185 (0.066) | 0.191 (0.067) | 0.199 (0.070) |
| high | uniform | 0.237 (0.060) | 0.238 (0.069) | 0.254 (0.063) | 0.265 (0.068) |

*Note.* Numbers presented are means of the sensitivities at a false positive rate of 5% across the 100 replications per condition (*SD*s in parentheses). Type of CR = type of careless responding; PR = conventional personal reliability (i.e., even-odd consistency); STDEV_RPR25 = standard deviation among the personal reliability values that were obtained when calculating the resampled personal reliability with 25 sets of scale half pairings; STDEV_RPR50 = standard deviation among the personal reliability values that were obtained when calculating the resampled personal reliability with 50 sets of scale half pairings; STDEV_RPR100 = standard deviation among the personal reliability values that were obtained when calculating the resampled personal reliability with 100 sets of scale half pairings; low = low item error per facet with normally distributed errors with *N*(0, 0.5) for all items of each factor; mediocre = mediocre item error per facet with normally distributed errors with *N*(0, 0.5) for one half of the items of each factor and normally distributed errors with *N*(0, 1.5) for the other half of the items of each factor; high = high item error per facet with normally distributed errors with *N*(0, 1.5) for all items of each factor; invariant = invariant careless responding; uniform = uniform random careless responding; Full careless responding = all item responses of the response protocol were replaced with simulated careless responses; Partial careless responding = 50% of the item responses of the response protocol were randomly selected and replaced with simulated careless responses.

^a^ Sensitivities could not be computed because of missing values in the matrix (i.e., non-zero sensitivities could only be calculated for false positive rates that were higher than the 5% level that we aimed for).

#### Table S6

*Cut-Off Values at a False Positive Rate of 5% for the Conventional Personal Reliability and Three Resampled Personal Reliability Versions Across Simulation Conditions*

| Error per facet | Type of CR | PR | STDEV_RPR25 | STDEV_RPR50 | STDEV_RPR100 |
| --- | --- | --- | --- | --- | --- |
| Full careless responding | | | | | |
| 5 facets in survey |  |  |  |  |  |
| low | invariant | -0.257 (0.249) | 0.563 (0.038) | 0.565 (0.034) | 0.565 (0.035) |
| low | uniform | -0.310 (0.246) | 0.570 (0.035) | 0.567 (0.032) | 0.568 (0.029) |
| mediocre | invariant | -1 (0) | 0.646 (0.021) | 0.635 (0.016) | 0.629 (0.015) |
| mediocre | uniform | -1 (0) | 0.649 (0.017) | 0.635 (0.015) | 0.628 (0.013) |
| high | invariant | -1 (0) | 0.684 (0.015) | 0.672 (0.015) | 0.662 (0.014) |
| high | uniform | -1 (0) | 0.686 (0.015) | 0.674 (0.015) | 0.666 (0.013) |
| 15 facets in survey |  |  |  |  |  |
| low | invariant | 0.515 (0.047) | 0.142 (0.011) | 0.142 (0.012) | 0.141 (0.012) |
| low | uniform | 0.518 (0.043) | 0.143 (0.012) | 0.141 (0.011) | 0.141 (0.011) |
| mediocre | invariant | -0.200 (0.095) | 0.308 (0.022) | 0.309 (0.020) | 0.307 (0.018) |
| mediocre | uniform | -0.209 (0.102) | 0.310 (0.021) | 0.310 (0.020) | 0.309 (0.019) |
| high | invariant | -0.647 (0.140) | 0.405 (0.014) | 0.401 (0.016) | 0.399 (0.013) |
| high | uniform | -0.640 (0.144) | 0.406 (0.017) | 0.401 (0.014) | 0.397 (0.014) |
| 30 facets in survey |  |  |  |  |  |
| low | invariant | 0.638 (0.020) | 0.076 (0.004) | 0.075 (0.004) | 0.074 (0.004) |
| low | uniform | 0.639 (0.023) | 0.076 (0.004) | 0.075 (0.004) | 0.075 (0.004) |
| mediocre | invariant | 0.095 (0.048) | 0.167 (0.008) | 0.165 (0.008) | 0.164 (0.008) |
| mediocre | uniform | 0.106 (0.051) | 0.168 (0.009) | 0.167 (0.008) | 0.165 (0.008) |
| high | invariant | -0.203 (0.073) | 0.252 (0.013) | 0.249 (0.012) | 0.247 (0.011) |
| high | uniform | -0.208 (0.077) | 0.251 (0.014) | 0.248 (0.012) | 0.247 (0.013) |
| Partial careless responding | | | | | |
| 5 facets in survey |  |  |  |  |  |
| low | invariant | -0.324 (0.291) | 0.566 (0.034) | 0.566 (0.036) | 0.567 (0.032) |
| low | uniform | -0.323 (0.274) | 0.572 (0.036) | 0.567 (0.036) | 0.571 (0.036) |
| mediocre | invariant | -1 (0) | 0.645 (0.018) | 0.635 (0.016) | 0.628 (0.015) |
| mediocre | uniform | -1 (0) | 0.647 (0.018) | 0.636 (0.014) | 0.630 (0.013) |
| high | invariant | -1 (0) | 0.684 (0.015) | 0.672 (0.014) | 0.665 (0.012) |
| high | uniform | -1 (0) | 0.684 (0.016) | 0.671 (0.015) | 0.663 (0.013) |
| 15 facets in survey |  |  |  |  |  |
| low | invariant | 0.522 (0.045) | 0.141 (0.012) | 0.141 (0.012) | 0.141 (0.012) |
| low | uniform | 0.514 (0.039) | 0.142 (0.011) | 0.141 (0.011) | 0.141 (0.011) |
| mediocre | invariant | -0.215 (0.101) | 0.310 (0.020) | 0.310 (0.020) | 0.307 (0.019) |
| mediocre | uniform | -0.227 (0.101) | 0.312 (0.021) | 0.314 (0.019) | 0.312 (0.017) |
| high | invariant | -0.627 (0.127) | 0.403 (0.018) | 0.398 (0.014) | 0.394 (0.015) |
| high | uniform | -0.656 (0.158) | 0.407 (0.014) | 0.402 (0.014) | 0.398 (0.013) |
| 30 facets in survey |  |  |  |  |  |
| low | invariant | 0.637 (0.021) | 0.076 (0.005) | 0.075 (0.004) | 0.074 (0.004) |
| low | uniform | 0.639 (0.018) | 0.076 (0.004) | 0.075 (0.004) | 0.075 (0.004) |
| mediocre | invariant | 0.095 (0.051) | 0.170 (0.009) | 0.166 (0.009) | 0.165 (0.009) |
| mediocre | uniform | 0.105 (0.054) | 0.167 (0.010) | 0.166 (0.010) | 0.165 (0.010) |
| high | invariant | -0.208 (0.081) | 0.251 (0.014) | 0.248 (0.013) | 0.246 (0.013) |
| high | uniform | -0.195 (0.065) | 0.249 (0.013) | 0.244 (0.011) | 0.242 (0.013) |

*Note.* Numbers presented are means of the cut-off values at a false positive rate of 5 % across the 100 replications per condition (*SD*s in parentheses). The standard deviation of the PR values does not have the same metric (i.e., it ranges from 0 to 1) as the conventional PR (i.e., ranging from -1 to 1). Hence the cut-off values cannot directly be compared between these two types of indices. Type of CR = type of careless responding; PR = conventional personal reliability (i.e., even-odd consistency); STDEV_RPR25 = standard deviation among the personal reliability values that were obtained when calculating the resampled personal reliability with 25 sets of scale half pairings; STDEV_RPR50 = standard deviation among the personal reliability values that were obtained when calculating the resampled personal reliability with 50 sets of scale half pairings; STDEV_RPR100 = standard deviation among the personal reliability values that were obtained when calculating the resampled personal reliability with 100 sets of scale half pairings; low = low item error per facet with normally distributed errors with *N*(0, 0.5) for all items of each factor; mediocre = mediocre item error per facet with normally distributed errors with *N*(0, 0.5) for one half of the items of each factor and normally distributed errors with *N*(0, 1.5) for the other half of the items of each factor; high = high item error per facet with normally distributed errors with *N*(0, 1.5) for all items of each factor; invariant = invariant careless responding; uniform = uniform random careless responding; Full careless responding = all item responses of the response protocol were replaced with simulated careless responses; Partial careless responding = 50% of the item responses of the response protocol were randomly selected and replaced with simulated careless responses.

## Replacing the Last 50% of Items by Careless Response Patterns in the Partial Careless Responding Condition

In contrast to the main paper, the results of the following three sections are based on replacing the last 50% of item with careless response patterns for the partial careless responding conditions. This represents the case when a fixed construct-wise item presentation mode is chosen.

### Mean-Based Resampled Personal Reliability Measures

Tables S7 to S9 show the results when the mean is used for computing RPR.

#### Table S7

*Area Under the Curve for the Conventional Personal Reliability and Three Resampled Personal Reliability Versions Across Simulation Conditions*

| Error per facet | Type of CR | PR | RPR25 | RPR50 | RPR100 |
| --- | --- | --- | --- | --- | --- |
| Full careless responding | | | | | |
| 5 facets in survey |  |  |  |  |  |
| low | invariant | 0.837 (0.024) | 0.904 (0.015) | 0.905 (0.015) | 0.906 (0.015) |
| low | uniform | 0.840 (0.027) | 0.905 (0.019) | 0.906 (0.019) | 0.906 (0.018) |
| mediocre | invariant | 0.655 (0.032) | 0.733 (0.030) | 0.734 (0.030) | 0.735 (0.030) |
| mediocre | uniform | 0.661 (0.033) | 0.730 (0.031) | 0.731 (0.031) | 0.732 (0.031) |
| high | invariant | 0.589 (0.033) | 0.658 (0.032) | 0.661 (0.032) | 0.661 (0.032) |
| high | uniform | 0.587 (0.032) | 0.642 (0.034) | 0.643 (0.034) | 0.644 (0.034) |
| 15 facets in survey |  |  |  |  |  |
| low | invariant | 0.981 (0.007) | 0.994 (0.003) | 0.995 (0.003) | 0.995 (0.003) |
| low | uniform | 0.981 (0.007) | 0.994 (0.003) | 0.995 (0.002) | 0.995 (0.003) |
| mediocre | invariant | 0.826 (0.028) | 0.879 (0.021) | 0.880 (0.021) | 0.881 (0.021) |
| mediocre | uniform | 0.827 (0.024) | 0.881 (0.020) | 0.883 (0.020) | 0.883 (0.020) |
| high | invariant | 0.713 (0.031) | 0.759 (0.029) | 0.760 (0.029) | 0.760 (0.029) |
| high | uniform | 0.716 (0.034) | 0.760 (0.032) | 0.761 (0.032) | 0.762 (0.032) |
| 30 facets in survey |  |  |  |  |  |
| low | invariant | 0.998 (0.001) | 1 (0) | 1 (0) | 1 (0) |
| low | uniform | 0.999 (0.001) | 1 (0) | 1 (0) | 1 (0) |
| mediocre | invariant | 0.914 (0.017) | 0.954 (0.010) | 0.955 (0.010) | 0.956 (0.010) |
| mediocre | uniform | 0.915 (0.016) | 0.954 (0.011) | 0.955 (0.011) | 0.956 (0.011) |
| high | invariant | 0.803 (0.027) | 0.849 (0.024) | 0.851 (0.023) | 0.852 (0.023) |
| high | uniform | 0.795 (0.031) | 0.846 (0.025) | 0.846 (0.026) | 0.846 (0.026) |
| Partial careless responding | | | | | |
| 5 facets in survey |  |  |  |  |  |
| low | invariant | 0.432 (0.040) | 0.515 (0.044) | 0.514 (0.043) | 0.514 (0.041) |
| low | uniform | 0.755 (0.032) | 0.834 (0.024) | 0.836 (0.025) | 0.836 (0.025) |
| mediocre | invariant | 0.355 (0.036) | 0.375 (0.039) | 0.376 (0.039) | 0.374 (0.040) |
| mediocre | uniform | 0.593 (0.039) | 0.647 (0.037) | 0.647 (0.038) | 0.648 (0.037) |
| high | invariant | 0.370 (0.035) | 0.380 (0.039) | 0.381 (0.037) | 0.381 (0.037) |
| high | uniform | 0.535 (0.034) | 0.572 (0.032) | 0.571 (0.032) | 0.571 (0.032) |
| 15 facets in survey |  |  |  |  |  |
| low | invariant | 0.365 (0.037) | 0.389 (0.044) | 0.388 (0.041) | 0.387 (0.041) |
| low | uniform | 0.924 (0.018) | 0.968 (0.010) | 0.969 (0.010) | 0.969 (0.010) |
| mediocre | invariant | 0.277 (0.033) | 0.262 (0.034) | 0.261 (0.034) | 0.260 (0.034) |
| mediocre | uniform | 0.700 (0.033) | 0.755 (0.029) | 0.757 (0.029) | 0.758 (0.029) |
| high | invariant | 0.299 (0.036) | 0.282 (0.035) | 0.281 (0.035) | 0.281 (0.035) |
| high | uniform | 0.604 (0.033) | 0.634 (0.033) | 0.635 (0.033) | 0.636 (0.033) |
| 30 facets in survey |  |  |  |  |  |
| low | invariant | 0.313 (0.037) | 0.287 (0.035) | 0.287 (0.035) | 0.286 (0.035) |
| low | uniform | 0.979 (0.008) | 0.995 (0.002) | 0.995 (0.002) | 0.995 (0.002) |
| mediocre | invariant | 0.222 (0.028) | 0.187 (0.027) | 0.186 (0.027) | 0.186 (0.027) |
| mediocre | uniform | 0.777 (0.029) | 0.832 (0.026) | 0.833 (0.026) | 0.834 (0.026) |
| high | invariant | 0.226 (0.030) | 0.193 (0.029) | 0.191 (0.029) | 0.191 (0.030) |
| high | uniform | 0.653 (0.036) | 0.684 (0.035) | 0.683 (0.034) | 0.685 (0.034) |

*Note.* Numbers presented are means of areas under the receiver operating characteristic curves across the 100 replications per condition (*SD*s in parentheses). Type of CR = type of careless responding; PR = conventional personal reliability (i.e., even-odd consistency); RPR25 = mean-based resampled personal reliability with 25 sets of scale half pairings; RPR50 = mean-based resampled personal reliability with 50 sets of scale half pairings; RPR100 = mean-based resampled personal reliability with 100 sets of scale half pairings; low = low item error per facet with normally distributed errors with *N*(0, 0.5) for all items of each factor; mediocre = mediocre item error per facet with normally distributed errors with *N*(0, 0.5) for one half of the items of each factor and normally distributed errors with *N*(0, 1.5) for the other half of the items of each factor; high = high item error per facet with normally distributed errors with *N*(0, 1.5) for all items of each factor; invariant = invariant careless responding; uniform = uniform random careless responding; Full careless responding = all item responses of the response protocol were replaced with simulated careless responses; Partial careless responding = the last 50% of the item responses of the response protocol were selected and replaced with simulated careless responses. Conditions in which the AUCs fell substantially below .5 are shown in gray. In these conditions the PR measures were more indicative of careful than for careless responding.

#### Table S8

*Sensitivity at a False Positive Rate of 5% for the Conventional Personal Reliability and Three Resampled Personal Reliability Versions Across Simulation Conditions*

| Error per facet | Type of CR | PR | RPR25 | RPR50 | RPR100 |
| --- | --- | --- | --- | --- | --- |
| Full careless responding | | | | | |
| 5 facets in survey |  |  |  |  |  |
| low | invariant | 0.397 (0.068) _[97]_ | 0.504 (0.078) | 0.504 (0.086) | 0.506 (0.088) |
| low | uniform | 0.434 (0.077) _[98]_ | 0.499 (0.100) | 0.505 (0.100) | 0.504 (0.099) |
| mediocre | invariant | ^a^ | 0.192 (0.048) | 0.187 (0.046) | 0.186 (0.047) |
| mediocre | uniform | ^a^ | 0.150 (0.054) | 0.154 (0.056) | 0.154 (0.056) |
| high | invariant | ^a^ | 0.175 (0.042) | 0.178 (0.042) | 0.180 (0.041) |
| high | uniform | ^a^ | 0.107 (0.042) | 0.105 (0.049) | 0.105 (0.041) |
| 15 facets in survey |  |  |  |  |  |
| low | invariant | 0.903 (0.040) | 0.979 (0.017) | 0.980 (0.016) | 0.980 (0.016) |
| low | uniform | 0.906 (0.038) | 0.978 (0.021) | 0.977 (0.021) | 0.976 (0.021) |
| mediocre | invariant | 0.373 (0.075) | 0.459 (0.086) | 0.455 (0.084) | 0.463 (0.084) |
| mediocre | uniform | 0.378 (0.082) | 0.474 (0.088) | 0.467 (0.089) | 0.478 (0.090) |
| high | invariant | 0.198 (0.056) _[99]_ | 0.226 (0.058) | 0.225 (0.064) | 0.227 (0.066) |
| high | uniform | 0.192 (0.057) _[99]_ | 0.232 (0.069) | 0.228 (0.072) | 0.232 (0.072) |
| 30 facets in survey |  |  |  |  |  |
| low | invariant | 0.994 (0.008) | 1 (0) | 0.9999 (0.0011) | 1 (0) |
| low | uniform | 0.996 (0.007) | 1 (0) | 0.9999 (0.0011) | 0.9999 (0.0011) |
| mediocre | invariant | 0.611 (0.080) | 0.747 (0.072) | 0.749 (0.071) | 0.754 (0.069) |
| mediocre | uniform | 0.622 (0.073) | 0.756 (0.067) | 0.760 (0.067) | 0.762 (0.068) |
| high | invariant | 0.324 (0.072) | 0.398 (0.083) | 0.400 (0.079) | 0.402 (0.081) |
| high | uniform | 0.313 (0.076) | 0.397 (0.087) | 0.396 (0.092) | 0.394 (0.09) |
| Partial careless responding | | | | | |
| 5 facets in survey |  |  |  |  |  |
| low | invariant | 0.041 (0.022) _[93]_ | 0.052 (0.033) | 0.052 (0.033) | 0.052 (0.032) |
| low | uniform | 0.302 (0.068) _[97]_ | 0.334 (0.080) | 0.328 (0.088) | 0.338 (0.085) |
| mediocre | invariant | ^a^ | 0.034 (0.017) | 0.033 (0.017) | 0.031 (0.015) |
| mediocre | uniform | ^a^ | 0.098 (0.039) | 0.099 (0.038) | 0.096 (0.040) |
| high | invariant | ^a^ | 0.037 (0.019) | 0.035 (0.019) | 0.036 (0.019) |
| high | uniform | ^a^ | 0.074 (0.034) | 0.074 (0.037) | 0.073 (0.033) |
| 15 facets in survey |  |  |  |  |  |
| low | invariant | 0.041 (0.020) _[96]_ | 0.050 (0.024) | 0.051 (0.024) | 0.052 (0.025) |
| low | uniform | 0.693 (0.065) | 0.836 (0.057) | 0.834 (0.061) | 0.837 (0.058) |
| mediocre | invariant | 0.027 (0.015) _[93]_ | 0.028 (0.016) | 0.027 (0.016) | 0.026 (0.015) |
| mediocre | uniform | 0.199 (0.056) | 0.235 (0.061) | 0.237 (0.061) | 0.240 (0.061) |
| high | invariant | 0.032 (0.017) _[93]_ | 0.029 (0.016) | 0.029 (0.015) | 0.028 (0.015) |
| high | uniform | 0.108 (0.041) | 0.117 (0.043) | 0.118 (0.044) | 0.115 (0.044) |
| 30 facets in survey |  |  |  |  |  |
| low | invariant | 0.046 (0.025) _[98]_ | 0.047 (0.023) | 0.048 (0.024) | 0.047 (0.022) |
| low | uniform | 0.900 (0.039) | 0.977 (0.018) | 0.977 (0.018) | 0.978 (0.018) |
| mediocre | invariant | 0.023 (0.013) _[93]_ | 0.023 (0.012) | 0.023 (0.012) | 0.023 (0.012) |
| mediocre | uniform | 0.312 (0.071) | 0.385 (0.075) | 0.388 (0.071) | 0.389 (0.074) |
| high | invariant | 0.023 (0.013) _[87]_ | 0.020 (0.010) | 0.021 (0.010) | 0.021 (0.01) |
| high | uniform | 0.144 (0.052) | 0.166 (0.058) | 0.165 (0.058) | 0.166 (0.061) |

*Note.* Numbers presented are means of the sensitivities at a false positive rate of 5% across the 100 replications per condition (*SD*s in parentheses). For some conditions, however, we could not achieve 100 successful replications even though we oversampled with 40 extra replications. For these conditions, the number of successful replications is shown in squared brackets and in lowercase. Type of CR = type of careless responding; PR = conventional personal reliability (i.e., even-odd consistency); RPR25 = mean-based resampled personal reliability with 25 sets of scale half pairings; RPR50 = mean-based resampled personal reliability with 50 sets of scale half pairings; RPR100 = mean-based resampled personal reliability with 100 sets of scale half pairings; low = low item error per facet with normally distributed errors with *N*(0, 0.5) for all items of each factor; mediocre = mediocre item error per facet with normally distributed errors with *N*(0, 0.5) for one half of the items of each factor and normally distributed errors with *N*(0, 1.5) for the other half of the items of each factor; high = high item error per facet with normally distributed errors with *N*(0, 1.5) for all items of each factor; invariant = invariant careless responding; uniform = uniform random careless responding; Full careless responding = all item responses of the response protocol were replaced with simulated careless responses; Partial careless responding = the last 50% of the item responses of the response protocol were selected and replaced with simulated careless responses. Conditions in which the AUCs fell substantially below .5 are shown in gray. In these conditions the PR measures were more indicative of careful than for careless responding.

^a^ Sensitivities could not be computed because of missing values in the matrix (i.e., non-zero sensitivities could only be calculated for false positive rates that were higher than the 5% level that we aimed for).

#### Table S9

*Cut-Off Values at a False Positive Rate of 5% for the Conventional Personal Reliability and Three Resampled Personal Reliability Versions Across Simulation Conditions*

| Error per facet | Type of CR | PR | RPR25 | RPR50 | RPR100 |
| --- | --- | --- | --- | --- | --- |
| Full careless responding | | | | | |
| 5 facets in survey |  |  |  |  |  |
| low | invariant | -0.389 (0.300) | -0.174 (0.140) | -0.172 (0.145) | -0.170 (0.147) |
| low | uniform | -0.314 (0.290) | -0.135 (0.171) | -0.121 (0.170) | -0.126 (0.172) |
| mediocre | invariant | -1 (0) | -0.787 (0.075) | -0.783 (0.066) | -0.771 (0.069) |
| mediocre | uniform | -1 (0) | -0.799 (0.065) | -0.788 (0.066) | -0.785 (0.069) |
| high | invariant | -1 (0) | -0.865 (0.050) | -0.852 (0.055) | -0.847 (0.052) |
| high | uniform | -1 (0) | -0.864 (0.050) | -0.857 (0.055) | -0.852 (0.048) |
| 15 facets in survey |  |  |  |  |  |
| low | invariant | 0.523 (0.042) | 0.566 (0.033) | 0.566 (0.031) | 0.568 (0.030) |
| low | uniform | 0.523 (0.042) | 0.568 (0.035) | 0.567 (0.036) | 0.567 (0.037) |
| mediocre | invariant | -0.216 (0.094) | -0.093 (0.083) | -0.093 (0.083) | -0.088 (0.083) |
| mediocre | uniform | -0.221 (0.112) | -0.089 (0.080) | -0.091 (0.083) | -0.086 (0.084) |
| high | invariant | -0.651 (0.138) | -0.454 (0.082) | -0.456 (0.082) | -0.449 (0.081) |
| high | uniform | -0.657 (0.136) | -0.454 (0.088) | -0.454 (0.086) | -0.450 (0.086) |
| 30 facets in survey |  |  |  |  |  |
| low | invariant | 0.637 (0.021) | 0.664 (0.016) | 0.664 (0.015) | 0.665 (0.016) |
| low | uniform | 0.641 (0.021) | 0.668 (0.016) | 0.669 (0.017) | 0.669 (0.016) |
| mediocre | invariant | 0.095 (0.050) | 0.168 (0.040) | 0.171 (0.039) | 0.172 (0.038) |
| mediocre | uniform | 0.102 (0.050) | 0.172 (0.041) | 0.175 (0.039) | 0.176 (0.039) |
| high | invariant | -0.215 (0.069) | -0.122 (0.059) | -0.118 (0.056) | -0.118 (0.057) |
| high | uniform | -0.212 (0.073) | -0.114 (0.060) | -0.113 (0.063) | -0.111 (0.062) |
| Partial careless responding | | | | | |
| 5 facets in survey |  |  |  |  |  |
| low | invariant | -0.296 (0.292) | -0.127 (0.170) | -0.122 (0.175) | -0.120 (0.172) |
| low | uniform | -0.340 (0.271) | -0.131 (0.156) | -0.135 (0.161) | -0.127 (0.157) |
| mediocre | invariant | -1 (0) | -0.784 (0.069) | -0.778 (0.063) | -0.774 (0.062) |
| mediocre | uniform | -1 (0) | -0.785 (0.069) | -0.779 (0.064) | -0.780 (0.066) |
| high | invariant | -1 (0) | -0.868 (0.046) | -0.861 (0.044) | -0.856 (0.046) |
| high | uniform | -1 (0) | -0.863 (0.049) | -0.855 (0.048) | -0.850 (0.047) |
| 15 facets in survey |  |  |  |  |  |
| low | invariant | 0.525 (0.039) | 0.564 (0.035) | 0.567 (0.032) | 0.570 (0.031) |
| low | uniform | 0.525 (0.037) | 0.569 (0.032) | 0.568 (0.035) | 0.569 (0.033) |
| mediocre | invariant | -0.208 (0.097) | -0.080 (0.087) | -0.083 (0.089) | -0.079 (0.087) |
| mediocre | uniform | -0.218 (0.102) | -0.090 (0.083) | -0.088 (0.084) | -0.083 (0.082) |
| high | invariant | -0.606 (0.130) | -0.421 (0.080) | -0.419 (0.082) | -0.420 (0.082) |
| high | uniform | -0.632 (0.146) | -0.436 (0.077) | -0.439 (0.086) | -0.438 (0.085) |
| 30 facets in survey |  |  |  |  |  |
| low | invariant | 0.641 (0.019) | 0.666 (0.017) | 0.667 (0.017) | 0.667 (0.017) |
| low | uniform | 0.641 (0.023) | 0.665 (0.016) | 0.666 (0.017) | 0.666 (0.017) |
| mediocre | invariant | 0.093 (0.049) | 0.175 (0.033) | 0.176 (0.036) | 0.177 (0.036) |
| mediocre | uniform | 0.103 (0.060) | 0.178 (0.040) | 0.180 (0.040) | 0.180 (0.039) |
| high | invariant | -0.198 (0.060) | -0.102 (0.053) | -0.102 (0.054) | -0.103 (0.052) |
| high | uniform | -0.205 (0.073) | -0.108 (0.060) | -0.106 (0.061) | -0.105 (0.061) |

*Note.* Numbers presented are means of the cut-off values at a false-positive rate of 5 % across the 100 replications per condition (*SD*s in parentheses). Type of CR = type of careless responding; PR = conventional personal reliability (i.e., even-odd consistency); RPR25 = mean-based resampled personal reliability with 25 sets of scale half pairings; RPR50 = mean-based resampled personal reliability with 50 sets of scale half pairings; RPR100 = mean-based resampled personal reliability with 100 sets of scale half pairings; low = low item error per facet with normally distributed errors with *N*(0, 0.5) for all items of each factor; mediocre = mediocre item error per facet with normally distributed errors with *N*(0, 0.5) for one half of the items of each factor and normally distributed errors with *N*(0, 1.5) for the other half of the items of each factor; high = high item error per facet with normally distributed errors with *N*(0, 1.5) for all items of each factor; invariant = invariant careless responding; uniform = uniform random careless responding; Full careless responding = all item responses of the response protocol were replaced with simulated careless responses; Partial careless responding = the last 50% of the item responses of the response protocol were selected and replaced with simulated careless responses. Conditions in which the AUCs fell substantially below .5 are shown in gray. In these conditions the PR measures were more indicative of careful than for careless responding. We therefore recommend not using the cut-off values that are displayed in gray.

### Median-Based Resampled Personal Reliability Measures

Tables S10 to S12 show the results when the median is used for computing the RPR.

#### Table S10

*Area Under the Curve for the Conventional Personal Reliability and Three Resampled Personal Reliability Versions Across Simulation Conditions*

| Error per facet | Type of CR | PR | RPR25 | RPR50 | RPR100 |
| --- | --- | --- | --- | --- | --- |
| Full careless responding | | | | | |
| 5 facets in survey |  |  |  |  |  |
| low | invariant | 0.837 (0.024) | 0.885 (0.020) | 0.886 (0.017) | 0.888 (0.019) |
| low | uniform | 0.840 (0.027) | 0.899 (0.021) | 0.901 (0.020) | 0.902 (0.019) |
| mediocre | invariant | 0.655 (0.032) | 0.698 (0.031) | 0.702 (0.031) | 0.703 (0.031) |
| mediocre | uniform | 0.661 (0.033) | 0.710 (0.032) | 0.714 (0.032) | 0.716 (0.032) |
| high | invariant | 0.589 (0.033) | 0.621 (0.032) | 0.625 (0.032) | 0.626 (0.034) |
| high | uniform | 0.587 (0.032) | 0.620 (0.034) | 0.623 (0.034) | 0.625 (0.034) |
| 15 facets in survey |  |  |  |  |  |
| low | invariant | 0.981 (0.007) | 0.994 (0.003) | 0.994 (0.003) | 0.995 (0.003) |
| low | uniform | 0.981 (0.007) | 0.994 (0.003) | 0.994 (0.003) | 0.995 (0.003) |
| mediocre | invariant | 0.826 (0.028) | 0.875 (0.022) | 0.876 (0.021) | 0.877 (0.021) |
| mediocre | uniform | 0.827 (0.024) | 0.877 (0.021) | 0.880 (0.020) | 0.881 (0.02) |
| high | invariant | 0.713 (0.031) | 0.757 (0.028) | 0.760 (0.029) | 0.759 (0.029) |
| high | uniform | 0.716 (0.034) | 0.758 (0.033) | 0.760 (0.032) | 0.761 (0.032) |
| 30 facets in survey |  |  |  |  |  |
| low | invariant | 0.998 (0.001) | 1 (0) | 1 (0) | 1 (0) |
| low | uniform | 0.999 (0.001) | 1 (0) | 1 (0) | 1 (0) |
| mediocre | invariant | 0.914 (0.017) | 0.952 (0.011) | 0.954 (0.010) | 0.955 (0.010) |
| mediocre | uniform | 0.915 (0.016) | 0.952 (0.011) | 0.954 (0.011) | 0.955 (0.011) |
| high | invariant | 0.803 (0.027) | 0.848 (0.024) | 0.850 (0.023) | 0.851 (0.023) |
| high | uniform | 0.795 (0.031) | 0.844 (0.025) | 0.845 (0.026) | 0.846 (0.026) |
| Partial careless responding | | | | | |
| 5 facets in survey |  |  |  |  |  |
| low | invariant | 0.432 (0.040) | 0.483 (0.048) | 0.481 (0.044) | 0.479 (0.041) |
| low | uniform | 0.755 (0.032) | 0.813 (0.026) | 0.818 (0.026) | 0.819 (0.025) |
| mediocre | invariant | 0.355 (0.036) | 0.358 (0.038) | 0.357 (0.040) | 0.354 (0.039) |
| mediocre | uniform | 0.593 (0.039) | 0.628 (0.038) | 0.627 (0.039) | 0.629 (0.038) |
| high | invariant | 0.370 (0.035) | 0.358 (0.038) | 0.359 (0.035) | 0.359 (0.037) |
| high | uniform | 0.535 (0.034) | 0.556 (0.031) | 0.556 (0.032) | 0.556 (0.032) |
| 15 facets in survey |  |  |  |  |  |
| low | invariant | 0.365 (0.037) | 0.378 (0.044) | 0.377 (0.041) | 0.375 (0.040) |
| low | uniform | 0.924 (0.018) | 0.964 (0.011) | 0.966 (0.011) | 0.966 (0.010) |
| mediocre | invariant | 0.277 (0.033) | 0.259 (0.033) | 0.257 (0.034) | 0.255 (0.034) |
| mediocre | uniform | 0.700 (0.033) | 0.747 (0.030) | 0.749 (0.030) | 0.750 (0.029) |
| high | invariant | 0.299 (0.036) | 0.277 (0.036) | 0.274 (0.034) | 0.274 (0.035) |
| high | uniform | 0.604 (0.033) | 0.633 (0.033) | 0.633 (0.034) | 0.635 (0.033) |
| 30 facets in survey |  |  |  |  |  |
| low | invariant | 0.313 (0.037) | 0.288 (0.035) | 0.287 (0.035) | 0.287 (0.034) |
| low | uniform | 0.979 (0.008) | 0.995 (0.003) | 0.995 (0.003) | 0.995 (0.002) |
| mediocre | invariant | 0.222 (0.028) | 0.186 (0.027) | 0.185 (0.027) | 0.184 (0.027) |
| mediocre | uniform | 0.777 (0.029) | 0.828 (0.026) | 0.829 (0.027) | 0.830 (0.027) |
| high | invariant | 0.226 (0.030) | 0.191 (0.029) | 0.188 (0.029) | 0.187 (0.029) |
| high | uniform | 0.653 (0.036) | 0.682 (0.034) | 0.682 (0.034) | 0.684 (0.034) |

*Note.* Numbers presented are means of areas under the receiver operating characteristic curves across the 100 replications per condition (*SD*s in parentheses). Type of CR = type of careless responding; PR = conventional personal reliability (i.e., even-odd consistency); RPR25 = median-based resampled personal reliability with 25 sets of scale half pairings; RPR50 = median-based resampled personal reliability with 50 sets of scale half pairings; RPR100 = median-based resampled personal reliability with 100 sets of scale half pairings; low = low item error per facet with normally distributed errors with *N*(0, 0.5) for all items of each factor; mediocre = mediocre item error per facet with normally distributed errors with *N*(0, 0.5) for one half of the items of each factor and normally distributed errors with *N*(0, 1.5) for the other half of the items of each factor; high = high item error per facet with normally distributed errors with *N*(0, 1.5) for all items of each factor; invariant = invariant careless responding; uniform = uniform random careless responding; Full careless responding = all item responses of the response protocol were replaced with simulated careless responses; Partial careless responding = the last 50% of the item responses of the response protocol were selected and replaced with simulated careless responses. Conditions in which the AUCs fell substantially below .5 are shown in gray. In these conditions the PR measures were more indicative of careful than for careless responding.

#### Table S11

*Sensitivity at a False Positive Rate of 5% for the Conventional Personal Reliability and Three Resampled Personal Reliability Versions Across Simulation Conditions*

| Error per facet | Type of CR | PR | RPR25 | RPR50 | RPR100 |
| --- | --- | --- | --- | --- | --- |
| Full careless responding | | | | | |
| 5 facets in survey |  |  |  |  |  |
| low | invariant | 0.397 (0.068) _[97]_ | 0.450 (0.080) | 0.454 (0.074) | 0.458 (0.068) |
| low | uniform | 0.434 (0.077) _[93]_ | 0.491 (0.089) | 0.500 (0.094) | 0.502 (0.094) |
| mediocre | invariant | ^a^ | 0.289 (0.047) _[2]_ | ^a^ | 0.322 (0) _[1]_ |
| mediocre | uniform | ^a^ | ^a^ | 0.3 (0) _[1]_ | 0.322 (0) _[1]_ |
| high | invariant | ^a^ | ^a^ | ^a^ | ^a^ |
| high | uniform | ^a^ | ^a^ | ^a^ | ^a^ |
| 15 facets in survey |  |  |  |  |  |
| low | invariant | 0.903 (0.040) | 0.976 (0.018) | 0.978 (0.017) | 0.978 (0.018) |
| low | uniform | 0.906 (0.038) | 0.975 (0.022) | 0.976 (0.021) | 0.975 (0.022) |
| mediocre | invariant | 0.373 (0.075) | 0.449 (0.088) | 0.447 (0.087) | 0.451 (0.084) |
| mediocre | uniform | 0.378 (0.082) | 0.462 (0.089) | 0.464 (0.087) | 0.471 (0.091) |
| high | invariant | 0.198 (0.056) _[99]_ | 0.226 (0.064) | 0.226 (0.068) | 0.227 (0.071) |
| high | uniform | 0.192 (0.057) _[99]_ | 0.233 (0.071) | 0.227 (0.075) | 0.232 (0.071) |
| 30 facets in survey |  |  |  |  |  |
| low | invariant | 0.994 (0.008) | 1 (0) | 0.9999 (0.001) | 1 (0) |
| low | uniform | 0.996 (0.007) | 1 (0) | 0.9999 (0.001) | 0.9999 (0.001) |
| mediocre | invariant | 0.611 (0.080) | 0.737 (0.068) | 0.747 (0.067) | 0.746 (0.066) |
| mediocre | uniform | 0.622 (0.073) | 0.749 (0.069) | 0.758 (0.069) | 0.757 (0.068) |
| high | invariant | 0.324 (0.072) | 0.398 (0.081) | 0.403 (0.080) | 0.402 (0.077) |
| high | uniform | 0.313 (0.076) | 0.390 (0.084) | 0.398 (0.094) | 0.394 (0.092) |
| Partial careless responding | | | | | |
| 5 facets in survey |  |  |  |  |  |
| low | invariant | 0.041 (0.022) _[93]_ | 0.046 (0.029) _[98]_ | 0.045 (0.029) _[99]_ | 0.043 (0.027) _[99]_ |
| low | uniform | 0.302 (0.068) _[97]_ | 0.321 (0.066) | 0.326 (0.070) _[99]_ | 0.325 (0.070) |
| mediocre | invariant | ^a^ | ^a^ | ^a^ | ^a^ |
| mediocre | uniform | ^a^ | ^a^ | ^a^ | 0.156 (0) _[1]_ |
| high | invariant | ^a^ | ^a^ | ^a^ | ^a^ |
| high | uniform | ^a^ | ^a^ | ^a^ | ^a^ |
| 15 facets in survey |  |  |  |  |  |
| low | invariant | 0.041 (0.020) _[96]_ | 0.050 (0.025) | 0.050 (0.026) | 0.051 (0.024) |
| low | uniform | 0.693 (0.065) | 0.819 (0.060) | 0.821 (0.059) | 0.827 (0.062) |
| mediocre | invariant | 0.027 (0.015) _[93]_ | 0.027 (0.015) _[97]_ | 0.026 (0.015) _[97]_ | 0.026 (0.015) _[99]_ |
| mediocre | uniform | 0.199 (0.056) | 0.225 (0.059) | 0.232 (0.059) | 0.231 (0.058) |
| high | invariant | 0.032 (0.017) _[93]_ | 0.028 (0.017) | 0.030 (0.016) | 0.029 (0.016) |
| high | uniform | 0.108 (0.041) | 0.115 (0.043) | 0.118 (0.044) | 0.118 (0.044) |
| 30 facets in survey |  |  |  |  |  |
| low | invariant | 0.046 (0.025) _[98]_ | 0.048 (0.023) | 0.048 (0.025) | 0.048 (0.023) |
| low | uniform | 0.900 (0.039) | 0.973 (0.020) | 0.974 (0.020) | 0.977 (0.019) |
| mediocre | invariant | 0.023 (0.013) _[93]_ | 0.022 (0.011) _[98]_ | 0.022 (0.011) _[99]_ | 0.023 (0.010) _[98]_ |
| mediocre | uniform | 0.312 (0.071) | 0.375 (0.074) | 0.379 (0.069) | 0.381 (0.074) |
| high | invariant | 0.023 (0.013) _[87]_ | 0.020 (0.010) _[95]_ | 0.020 (0.010) _[92]_ | 0.021 (0.010) _[91]_ |
| high | uniform | 0.144 (0.052) | 0.166 (0.058) | 0.164 (0.056) | 0.166 (0.060) |

*Note.* Numbers presented are means of the sensitivities at a false positive rate of 5% across the 100 replications per condition (*SD*s in parentheses). For some conditions, however, we could not achieve 100 successful replications even though we oversampled with 40 extra replications. For these conditions, the number of successful replications is shown in squared brackets and in lowercase. Type of CR = type of careless responding; PR = conventional personal reliability (i.e., even-odd consistency); RPR25 = median-based resampled personal reliability with 25 sets of scale half pairings; RPR50 = median-based resampled personal reliability with 50 sets of scale half pairings; RPR100 = median-based resampled personal reliability with 100 sets of scale half pairings; low = low item error per facet with normally distributed errors with *N*(0, 0.5) for all items of each factor; mediocre = mediocre item error per facet with normally distributed errors with *N*(0, 0.5) for one half of the items of each factor and normally distributed errors with *N*(0, 1.5) for the other half of the items of each factor; high = high item error per facet with normally distributed errors with *N*(0, 1.5) for all items of each factor; invariant = invariant careless responding; uniform = uniform random careless responding; Full careless responding = all item responses of the response protocol were replaced with simulated careless responses; Partial careless responding = the last 50% of the item responses of the response protocol were selected and replaced with simulated careless responses. Conditions in which the AUCs fell substantially below .5 are shown in gray. In these conditions the PR measures were more indicative of careful than for careless responding.

^a^ Sensitivities could not be computed because of missing values in the matrix (i.e., non-zero sensitivities could only be calculated for false positive rates that were higher than the 5% level that we aimed for).

#### Table S12

*Cut-Off Values at a False Positive Rate of 5% for the Conventional Personal Reliability and Three Resampled Personal Reliability Versions Across Simulation Conditions*

| Error per facet | Type of CR | PR | RPR25 | RPR50 | RPR100 |
| --- | --- | --- | --- | --- | --- |
| Full careless responding | | | | | |
| 5 facets in survey |  |  |  |  |  |
| low | invariant | -0.389 (0.300) | -0.167 (0.223) | -0.147 (0.222) | -0.157 (0.223) |
| low | uniform | -0.314 (0.290) | -0.134 (0.244) | -0.110 (0.241) | -0.098 (0.237) |
| mediocre | invariant | -1 (0) | -0.9997 (0.002) | -1 (0) | -0.9999 (0.001) |
| mediocre | uniform | -1 (0) | -1 (0) | -0.9999 (0.001) | -0.999 (0.01) |
| high | invariant | -1 (0) | -1 (0) | -1 (0) | -1 (0) |
| high | uniform | -1 (0) | -1 (0) | -1 (0) | -1 (0) |
| 15 facets in survey |  |  |  |  |  |
| low | invariant | 0.523 (0.042) | 0.573 (0.033) | 0.575 (0.029) | 0.576 (0.030) |
| low | uniform | 0.523 (0.042) | 0.575 (0.036) | 0.575 (0.036) | 0.577 (0.036) |
| mediocre | invariant | -0.216 (0.094) | -0.082 (0.084) | -0.083 (0.084) | -0.074 (0.084) |
| mediocre | uniform | -0.221 (0.112) | -0.081 (0.083) | -0.076 (0.082) | -0.075 (0.087) |
| high | invariant | -0.651 (0.138) | -0.472 (0.107) | -0.469 (0.104) | -0.464 (0.104) |
| high | uniform | -0.657 (0.136) | -0.473 (0.117) | -0.474 (0.115) | -0.469 (0.115) |
| 30 facets in survey |  |  |  |  |  |
| low | invariant | 0.637 (0.021) | 0.666 (0.016) | 0.666 (0.015) | 0.667 (0.016) |
| low | uniform | 0.641 (0.021) | 0.671 (0.016) | 0.671 (0.017) | 0.671 (0.016) |
| mediocre | invariant | 0.095 (0.050) | 0.171 (0.038) | 0.176 (0.038) | 0.175 (0.039) |
| mediocre | uniform | 0.102 (0.050) | 0.175 (0.040) | 0.178 (0.039) | 0.179 (0.039) |
| high | invariant | -0.215 (0.069) | -0.116 (0.059) | -0.11 (0.058) | -0.110 (0.057) |
| high | uniform | -0.212 (0.073) | -0.106 (0.062) | -0.102 (0.065) | -0.102 (0.063) |
| Partial careless responding | | | | | |
| 5 facets in survey |  |  |  |  |  |
| low | invariant | -0.296 (0.292) | -0.121 (0.229) | -0.111 (0.231) | -0.098 (0.238) |
| low | uniform | -0.340 (0.271) | -0.114 (0.240) | -0.111 (0.231) | -0.105 (0.230) |
| mediocre | invariant | -1 (0) | -1 (0) | -1 (0) | -1 (0) |
| mediocre | uniform | -1 (0) | -1 (0) | -1 (0) | -0.999 (0.005) |
| high | invariant | -1 (0) | -1 (0) | -1 (0) | -1 (0) |
| high | uniform | -1 (0) | -1 (0) | -1 (0) | -1 (0) |
| 15 facets in survey |  |  |  |  |  |
| low | invariant | 0.525 (0.039) | 0.572 (0.034) | 0.576 (0.033) | 0.579 (0.031) |
| low | uniform | 0.525 (0.037) | 0.577 (0.032) | 0.576 (0.033) | 0.579 (0.033) |
| mediocre | invariant | -0.208 (0.097) | -0.073 (0.087) | -0.072 (0.089) | -0.065 (0.088) |
| mediocre | uniform | -0.218 (0.102) | -0.083 (0.088) | -0.074 (0.084) | -0.071 (0.084) |
| high | invariant | -0.606 (0.130) | -0.434 (0.104) | -0.430 (0.099) | -0.430 (0.098) |
| high | uniform | -0.632 (0.146) | -0.46 (0.105) | -0.455 (0.109) | -0.447 (0.109) |
| 30 facets in survey |  |  |  |  |  |
| low | invariant | 0.641 (0.019) | 0.669 (0.016) | 0.670 (0.017) | 0.670 (0.017) |
| low | uniform | 0.641 (0.023) | 0.668 (0.017) | 0.668 (0.017) | 0.669 (0.016) |
| mediocre | invariant | 0.093 (0.049) | 0.176 (0.034) | 0.179 (0.036) | 0.180 (0.036) |
| mediocre | uniform | 0.103 (0.060) | 0.180 (0.040) | 0.184 (0.04) | 0.184 (0.039) |
| high | invariant | -0.198 (0.060) | -0.095 (0.056) | -0.095 (0.054) | -0.094 (0.052) |
| high | uniform | -0.205 (0.073) | -0.102 (0.061) | -0.098 (0.061) | -0.095 (0.06) |

*Note.* Numbers presented are means of the cut-off values at a false positive rate of 5% across the 100 replications per condition (*SD*s in parentheses). Type of CR = type of careless responding; PR = conventional personal reliability (i.e., even-odd consistency); RPR25 = median-based resampled personal reliability with 25 sets of scale half pairings; RPR50 = median-based resampled personal reliability with 50 sets of scale half pairings; RPR100 = median-based resampled personal reliability with 100 sets of scale half pairings; low = low item error per facet with normally distributed errors with *N*(0, 0.5) for all items of each factor; mediocre = mediocre item error per facet with normally distributed errors with *N*(0, 0.5) for one half of the items of each factor and normally distributed errors with *N*(0, 1.5) for the other half of the items of each factor; high = high item error per facet with normally distributed errors with *N*(0, 1.5) for all items of each factor; invariant = invariant careless responding; uniform = uniform random careless responding; Full careless responding = all item responses of the response protocol were replaced with simulated careless responses; Partial careless responding = the last 50% of the item responses of the response protocol were selected and replaced with simulated careless responses. Conditions in which the AUCs fell substantially below .5 are shown in gray. In these conditions the PR measures were more indicative of careful than for careless responding. We therefore recommend not using the cut-off values that are displayed in gray.

### Standard Deviation of the Personal Reliabilities as Detection Measure

Tables S13 to S15 show the results when the standard deviation of the PR values is used as detection measure.

#### Table S13

*Area Under the Curve for the Conventional Personal Reliability and Three Resampled Personal Reliability Versions Across Simulation Conditions*

| Error per facet | Type of CR | PR | STDEV_RPR25 | STDEV_RPR50 | STDEV_RPR100 |
| --- | --- | --- | --- | --- | --- |
| Full careless responding | | | | | |
| 5 facets in survey |  |  |  |  |  |
| low | invariant | 0.837 (0.024) | 0.774 (0.033) | 0.781 (0.034) | 0.79 (0.0320) |
| low | uniform | 0.840 (0.027) | 0.851 (0.023) | 0.861 (0.024) | 0.866 (0.022) |
| mediocre | invariant | 0.655 (0.032) | 0.608 (0.036) | 0.615 (0.036) | 0.618 (0.034) |
| mediocre | uniform | 0.661 (0.033) | 0.686 (0.034) | 0.691 (0.033) | 0.694 (0.034) |
| high | invariant | 0.589 (0.033) | 0.526 (0.041) | 0.529 (0.041) | 0.529 (0.042) |
| high | uniform | 0.587 (0.032) | 0.583 (0.034) | 0.587 (0.034) | 0.591 (0.034) |
| 15 facets in survey |  |  |  |  |  |
| low | invariant | 0.981 (0.007) | 0.983 (0.010) | 0.987 (0.008) | 0.989 (0.006) |
| low | uniform | 0.981 (0.007) | 0.982 (0.009) | 0.984 (0.008) | 0.986 (0.007) |
| mediocre | invariant | 0.826 (0.028) | 0.868 (0.023) | 0.875 (0.021) | 0.880 (0.020) |
| mediocre | uniform | 0.827 (0.024) | 0.868 (0.023) | 0.876 (0.023) | 0.879 (0.023) |
| high | invariant | 0.713 (0.031) | 0.711 (0.036) | 0.716 (0.035) | 0.720 (0.034) |
| high | uniform | 0.716 (0.034) | 0.710 (0.030) | 0.715 (0.034) | 0.718 (0.033) |
| 30 facets in survey |  |  |  |  |  |
| low | invariant | 0.998 (0.001) | 0.998 (0.003) | 0.999 (0.003) | 0.999 (0.002) |
| low | uniform | 0.999 (0.001) | 0.998 (0.004) | 0.999 (0.003) | 0.999 (0.002) |
| mediocre | invariant | 0.914 (0.017) | 0.951 (0.013) | 0.959 (0.010) | 0.962 (0.010) |
| mediocre | uniform | 0.915 (0.016) | 0.949 (0.013) | 0.956 (0.013) | 0.959 (0.011) |
| high | invariant | 0.803 (0.027) | 0.810 (0.027) | 0.823 (0.026) | 0.828 (0.025) |
| high | uniform | 0.795 (0.031) | 0.805 (0.029) | 0.814 (0.028) | 0.822 (0.027) |
| Partial careless responding | | | | | |
| 5 facets in survey |  |  |  |  |  |
| low | invariant | 0.432 (0.040) | 0.547 (0.040) | 0.545 (0.039) | 0.545 (0.039) |
| low | uniform | 0.755 (0.032) | 0.820 (0.027) | 0.826 (0.025) | 0.830 (0.024) |
| mediocre | invariant | 0.355 (0.036) | 0.418 (0.037) | 0.418 (0.038) | 0.416 (0.038) |
| mediocre | uniform | 0.593 (0.039) | 0.644 (0.039) | 0.648 (0.039) | 0.651 (0.039) |
| high | invariant | 0.370 (0.035) | 0.432 (0.042) | 0.433 (0.040) | 0.432 (0.042) |
| high | uniform | 0.535 (0.034) | 0.550 (0.034) | 0.552 (0.035) | 0.552 (0.035) |
| 15 facets in survey |  |  |  |  |  |
| low | invariant | 0.365 (0.037) | 0.449 (0.045) | 0.449 (0.042) | 0.446 (0.042) |
| low | uniform | 0.924 (0.018) | 0.972 (0.008) | 0.974 (0.009) | 0.975 (0.009) |
| mediocre | invariant | 0.277 (0.033) | 0.343 (0.036) | 0.339 (0.038) | 0.338 (0.037) |
| mediocre | uniform | 0.700 (0.033) | 0.790 (0.026) | 0.795 (0.025) | 0.798 (0.026) |
| high | invariant | 0.299 (0.036) | 0.381 (0.039) | 0.379 (0.038) | 0.378 (0.039) |
| high | uniform | 0.604 (0.033) | 0.625 (0.031) | 0.628 (0.031) | 0.631 (0.031) |
| 30 facets in survey |  |  |  |  |  |
| low | invariant | 0.313 (0.037) | 0.312 (0.036) | 0.305 (0.037) | 0.303 (0.037) |
| low | uniform | 0.979 (0.008) | 0.996 (0.002) | 0.997 (0.002) | 0.997 (0.002) |
| mediocre | invariant | 0.222 (0.028) | 0.285 (0.033) | 0.278 (0.034) | 0.276 (0.033) |
| mediocre | uniform | 0.777 (0.029) | 0.874 (0.021) | 0.882 (0.022) | 0.887 (0.021) |
| high | invariant | 0.226 (0.030) | 0.335 (0.034) | 0.330 (0.035) | 0.326 (0.036) |
| high | uniform | 0.653 (0.036) | 0.674 (0.033) | 0.681 (0.032) | 0.685 (0.032) |

*Note.* Numbers presented are means of areas under the receiver operating characteristic curves across the 100 replications per condition (*SD*s in parentheses). Type of CR = type of careless responding; PR = conventional personal reliability (i.e., even-odd consistency); STDEV_RPR25 = standard deviation among the personal reliability values that were obtained when calculating the resampled personal reliability with 25 sets of scale half pairings; STDEV_RPR50 = standard deviation among the personal reliability values that were obtained when calculating the resampled personal reliability with 50 sets of scale half pairings; STDEV_RPR100 = standard deviation among the personal reliability values that were obtained when calculating the resampled personal reliability with 100 sets of scale half pairings; low = low item error per facet with normally distributed errors with *N*(0, 0.5) for all items of each factor; mediocre = mediocre item error per facet with normally distributed errors with *N*(0, 0.5) for one half of the items of each factor and normally distributed errors with *N*(0, 1.5) for the other half of the items of each factor; high = high item error per facet with normally distributed errors with *N*(0, 1.5) for all items of each factor; invariant = invariant careless responding; uniform = uniform random careless responding; Full careless responding = all item responses of the response protocol were replaced with simulated careless responses; Partial careless responding = the last 50% of the item responses of the response protocol were selected and replaced with simulated careless responses. Conditions in which the AUCs fell substantially below .5 are shown in gray. In these conditions the PR measures were more indicative of careful than for careless responding.

#### Table S14

*Sensitivity at a False Positive Rate of 5% for the Conventional Personal Reliability and Three Resampled Personal Reliability Versions Across Simulation Conditions*

| Error per facet | Type of CR | PR | STDEV_RPR25 | STDEV_RPR50 | STDEV_RPR100 |
| --- | --- | --- | --- | --- | --- |
| Full careless responding | | | | | |
| 5 facets in survey |  |  |  |  |  |
| low | invariant | 0.397 (0.068) _[97]_ | 0.295 (0.084) | 0.301 (0.088) | 0.292 (0.084) |
| low | uniform | 0.434 (0.077) _[98]_ | 0.307 (0.095) | 0.316 (0.102) | 0.313 (0.106) |
| mediocre | invariant | ^a^ | 0.137 (0.047) | 0.149 (0.044) | 0.157 (0.055) |
| mediocre | uniform | ^a^ | 0.139 (0.052) | 0.143 (0.053) | 0.146 (0.050) |
| high | invariant | ^a^ | 0.071 (0.030) | 0.072 (0.033) | 0.075 (0.031) _[99]_ |
| high | uniform | ^a^ | 0.074 (0.032) | 0.075 (0.032) | 0.078 (0.034) |
| 15 facets in survey |  |  |  |  |  |
| low | invariant | 0.903 (0.040) | 0.943 (0.027) | 0.951 (0.028) | 0.957 (0.026) |
| low | uniform | 0.906 (0.038) | 0.936 (0.033) | 0.946 (0.030) | 0.948 (0.030) |
| mediocre | invariant | 0.373 (0.075) | 0.437 (0.091) | 0.462 (0.086) | 0.476 (0.090) |
| mediocre | uniform | 0.378 (0.082) | 0.452 (0.083) | 0.473 (0.089) | 0.475 (0.087) |
| high | invariant | 0.198 (0.056) _[99]_ | 0.140 (0.058) | 0.134 (0.057) | 0.141 (0.059) |
| high | uniform | 0.192 (0.057) _[99]_ | 0.135 (0.044) | 0.137 (0.053) | 0.141 (0.053) |
| 30 facets in survey |  |  |  |  |  |
| low | invariant | 0.994 (0.008) | 0.996 (0.006) | 0.998 (0.004) | 0.999 (0.004) |
| low | uniform | 0.996 (0.007) | 0.996 (0.006) | 0.998 (0.005) | 0.998 (0.005) |
| mediocre | invariant | 0.611 (0.080) | 0.751 (0.073) | 0.776 (0.062) | 0.799 (0.063) |
| mediocre | uniform | 0.622 (0.073) | 0.756 (0.063) | 0.783 (0.060) | 0.800 (0.055) |
| high | invariant | 0.324 (0.072) | 0.311 (0.078) | 0.326 (0.075) | 0.343 (0.084) |
| high | uniform | 0.313 (0.076) | 0.319 (0.083) | 0.324 (0.086) | 0.345 (0.084) |
| Partial careless responding | | | | | |
| 5 facets in survey |  |  |  |  |  |
| low | invariant | 0.041 (0.022) _[93]_ | 0.074 (0.032) | 0.073 (0.033) | 0.074 (0.033) |
| low | uniform | 0.302 (0.068) _[97]_ | 0.312 (0.071) | 0.316 (0.075) | 0.330 (0.074) |
| mediocre | invariant | ^a^ | 0.071 (0.027) _[99]_ | 0.076 (0.030) | 0.076 (0.028) |
| mediocre | uniform | ^a^ | 0.135 (0.044) | 0.147 (0.049) | 0.149 (0.044) |
| high | invariant | ^a^ | 0.084 (0.034) | 0.097 (0.035) | 0.095 (0.035) |
| high | uniform | ^a^ | 0.073 (0.031) _[99]_ | 0.071 (0.032) | 0.069 (0.033) |
| 15 facets in survey |  |  |  |  |  |
| low | invariant | 0.041 (0.020) _[96]_ | 0.064 (0.028) _[98]_ | 0.068 (0.031) | 0.066 (0.03) |
| low | uniform | 0.693 (0.065) | 0.862 (0.044) | 0.875 (0.041) | 0.876 (0.046) |
| mediocre | invariant | 0.027 (0.015) _[93]_ | 0.046 (0.022) _[98]_ | 0.044 (0.022) _[99]_ | 0.046 (0.021) |
| mediocre | uniform | 0.199 (0.056) | 0.327 (0.069) | 0.342 (0.062) | 0.336 (0.067) |
| high | invariant | 0.032 (0.017) _[93]_ | 0.065 (0.027) _[98]_ | 0.066 (0.028) | 0.069 (0.028) |
| high | uniform | 0.108 (0.041) | 0.106 (0.043) | 0.111 (0.043) | 0.112 (0.044) |
| 30 facets in survey |  |  |  |  |  |
| low | invariant | 0.046 (0.025) _[98]_ | 0.044 (0.024) _[96]_ | 0.047 (0.024) _[97]_ | 0.047 (0.024) _[98]_ |
| low | uniform | 0.900 (0.039) | 0.979 (0.017) | 0.985 (0.013) | 0.985 (0.013) |
| mediocre | invariant | 0.023 (0.013) _[93]_ | 0.042 (0.021) | 0.043 (0.020) | 0.043 (0.021) |
| mediocre | uniform | 0.312 (0.071) | 0.514 (0.075) | 0.537 (0.078) | 0.551 (0.075) |
| high | invariant | 0.023 (0.013) _[87]_ | 0.053 (0.020) _[99]_ | 0.050 (0.019) _[99]_ | 0.052 (0.019) _[99]_ |
| high | uniform | 0.144 (0.052) | 0.156 (0.054) | 0.159 (0.058) | 0.168 (0.055) |

*Note.* Numbers presented are means of the sensitivities at a false positive rate of 5% across the 100 replications per condition (*SD*s in parentheses). For some conditions, however, we could not achieve 100 successful replications even though we oversampled with 40 extra replications. For these conditions, the number of successful replications is displayed in squared brackets and in lowercase. Type of CR = type of careless responding; PR = conventional personal reliability (i.e., even-odd consistency); STDEV_RPR25 = standard deviation among the personal reliability values that were obtained when calculating the resampled personal reliability with 25 sets of scale half pairings; STDEV_RPR50 = standard deviation among the personal reliability values that were obtained when calculating the resampled personal reliability with 50 sets of scale half pairings; STDEV_RPR100 = standard deviation among the personal reliability values that were obtained when calculating the resampled personal reliability with 100 sets of scale half pairings; low = low item error per facet with normally distributed errors with *N*(0, 0.5) for all items of each factor; mediocre = mediocre item error per facet with normally distributed errors with *N*(0, 0.5) for one half of the items of each factor and normally distributed errors with *N*(0, 1.5) for the other half of the items of each factor; high = high item error per facet with normally distributed errors with *N*(0, 1.5) for all items of each factor; invariant = invariant careless responding; uniform = uniform random careless responding; Full careless responding = all item responses of the response protocol were replaced with simulated careless responses; Partial careless responding = the last 50% of the item responses of the response protocol were selected and replaced with simulated careless responses. Conditions in which the AUCs fell substantially below .5 are shown in gray. In these conditions the PR measures were more indicative of careful than for careless responding.

^a^ Sensitivities could not be computed because of missing values in the matrix (i.e., non-zero sensitivities could only be calculated for false positive rates that were higher than the 5% level that we aimed for).

#### Table S15

*Cut-Off Values at a False Positive Rate of 5% for the Conventional Personal Reliability and Three Resampled Personal Reliability Versions Across Simulation Conditions*

| Error per facet | Type of CR | PR | STDEV_RPR25 | STDEV_RPR50 | STDEV_RPR100 |
| --- | --- | --- | --- | --- | --- |
| Full careless responding | | | | | |
| 5 facets in survey |  |  |  |  |  |
| low | invariant | -0.389 (0.300) | 0.571 (0.038) | 0.569 (0.034) | 0.571 (0.032) |
| low | uniform | -0.314 (0.290) | 0.571 (0.036) | 0.568 (0.036) | 0.568 (0.033) |
| mediocre | invariant | -1 (0) | 0.646 (0.017) | 0.633 (0.015) | 0.629 (0.013) |
| mediocre | uniform | -1 (0) | 0.644 (0.019) | 0.634 (0.016) | 0.628 (0.013) |
| high | invariant | -1 (0) | 0.686 (0.015) | 0.673 (0.011) | 0.665 (0.012) |
| high | uniform | -1 (0) | 0.684 (0.015) | 0.670 (0.014) | 0.663 (0.012) |
| 15 facets in survey |  |  |  |  |  |
| low | invariant | 0.523 (0.042) | 0.142 (0.011) | 0.141 (0.011) | 0.140 (0.011) |
| low | uniform | 0.523 (0.042) | 0.142 (0.012) | 0.142 (0.011) | 0.141 (0.012) |
| mediocre | invariant | -0.216 (0.094) | 0.314 (0.021) | 0.310 (0.019) | 0.309 (0.02) |
| mediocre | uniform | -0.221 (0.112) | 0.312 (0.020) | 0.309 (0.021) | 0.311 (0.021) |
| high | invariant | -0.651 (0.138) | 0.406 (0.017) | 0.402 (0.013) | 0.399 (0.014) |
| high | uniform | -0.657 (0.136) | 0.407 (0.013) | 0.402 (0.014) | 0.400 (0.012) |
| 30 facets in survey |  |  |  |  |  |
| low | invariant | 0.637 (0.021) | 0.076 (0.004) | 0.075 (0.004) | 0.075 (0.004) |
| low | uniform | 0.641 (0.021) | 0.076 (0.003) | 0.075 (0.003) | 0.074 (0.004) |
| mediocre | invariant | 0.095 (0.050) | 0.169 (0.009) | 0.168 (0.009) | 0.166 (0.009) |
| mediocre | uniform | 0.102 (0.050) | 0.168 (0.008) | 0.166 (0.008) | 0.165 (0.008) |
| high | invariant | -0.215 (0.069) | 0.253 (0.013) | 0.249 (0.012) | 0.248 (0.013) |
| high | uniform | -0.212 (0.073) | 0.250 (0.014) | 0.249 (0.013) | 0.247 (0.012) |
| Partial careless responding | | | | | |
| 5 facets in survey |  |  |  |  |  |
| low | invariant | -0.296 (0.292) | 0.571 (0.038) | 0.566 (0.033) | 0.566 (0.035) |
| low | uniform | -0.340 (0.271) | 0.570 (0.037) | 0.570 (0.036) | 0.567 (0.033) |
| mediocre | invariant | -1 (0) | 0.647 (0.017) | 0.634 (0.014) | 0.629 (0.013) |
| mediocre | uniform | -1 (0) | 0.648 (0.018) | 0.633 (0.015) | 0.627 (0.014) |
| high | invariant | -1 (0) | 0.684 (0.015) | 0.671 (0.013) | 0.664 (0.012) |
| high | uniform | -1 (0) | 0.685 (0.016) | 0.675 (0.013) | 0.665 (0.013) |
| 15 facets in survey |  |  |  |  |  |
| low | invariant | 0.525 (0.039) | 0.142 (0.011) | 0.140 (0.010) | 0.141 (0.011) |
| low | uniform | 0.525 (0.037) | 0.142 (0.011) | 0.140 (0.010) | 0.140 (0.012) |
| mediocre | invariant | -0.208 (0.097) | 0.307 (0.019) | 0.304 (0.019) | 0.306 (0.020) |
| mediocre | uniform | -0.218 (0.102) | 0.310 (0.019) | 0.307 (0.017) | 0.308 (0.019) |
| high | invariant | -0.606 (0.130) | 0.405 (0.017) | 0.399 (0.014) | 0.396 (0.013) |
| high | uniform | -0.632 (0.146) | 0.406 (0.015) | 0.399 (0.014) | 0.397 (0.012) |
| 30 facets in survey |  |  |  |  |  |
| low | invariant | 0.641 (0.019) | 0.076 (0.004) | 0.075 (0.004) | 0.075 (0.004) |
| low | uniform | 0.641 (0.023) | 0.076 (0.004) | 0.075 (0.004) | 0.074 (0.004) |
| mediocre | invariant | 0.093 (0.049) | 0.169 (0.009) | 0.166 (0.009) | 0.165 (0.008) |
| mediocre | uniform | 0.103 (0.060) | 0.168 (0.009) | 0.165 (0.008) | 0.165 (0.008) |
| high | invariant | -0.198 (0.060) | 0.247 (0.013) | 0.246 (0.012) | 0.243 (0.011) |
| high | uniform | -0.205 (0.073) | 0.249 (0.013) | 0.246 (0.013) | 0.244 (0.012) |

*Note.* Numbers presented are means of the cut-off values at a false positive rate of 5% across the 100 replications per condition (*SD*s in parentheses). The standard deviation of the PR values does not have the same metric (i.e., it ranges from 0 to 1) as the conventional PR (i.e., ranging from -1 to 1). Hence the cut-off values cannot directly be compared between these two types of indices. Type of CR = type of careless responding; PR = conventional personal reliability (i.e., even-odd consistency); STDEV_RPR25 = standard deviation among the personal reliability values that were obtained when calculating the resampled personal reliability with 25 sets of scale half pairings; STDEV_RPR50 = standard deviation among the personal reliability values that were obtained when calculating the resampled personal reliability with 50 sets of scale half pairings; STDEV_RPR100 = standard deviation among the personal reliability values that were obtained when calculating the resampled personal reliability with 100 sets of scale half pairings; low = low item error per facet with normally distributed errors with *N*(0, 0.5) for all items of each factor; mediocre = mediocre item error per facet with normally distributed errors with *N*(0, 0.5) for one half of the items of each factor and normally distributed errors with *N*(0, 1.5) for the other half of the items of each factor; high = high item error per facet with normally distributed errors with *N*(0, 1.5) for all items of each factor; invariant = invariant careless responding; uniform = uniform random careless responding; Full careless responding = all item responses of the response protocol were replaced with simulated careless responses; Partial careless responding = the last 50% of the item responses of the response protocol were selected and replaced with simulated careless responses. Conditions in which the AUCs fell substantially below .5 are shown in gray. In these conditions the PR measures were more indicative of careful than for careless responding. We therefore recommend not using the cut-off values that are displayed in gray.
